# ﻿Species diversity of bdelloid rotifer (Rotifera, Bdelloidea) in different areas in China, with a description of two new species

**DOI:** 10.3897/zookeys.1229.113385

**Published:** 2025-02-24

**Authors:** Wenbo Wang, Yufeng Yang, Aydin Örstan, Zhili He, Qing Wang

**Affiliations:** 1 Institute of Hydrobiology, Key Laboratory of Philosophy and Social Science in Guangdong Province, Jinan University/Southern Marine Science and Engineering Guangdong Laboratory (Zhuhai), Guangzhou 510632, China Jinan University/Southern Marine Science and Engineering Guangdong Laboratory (Zhuhai) Guangzhou China; 2 Research Associate, Section of Mollusks, Carnegie Museum of Natural History, Pittsburgh, Pennsylvania, USA Section of Mollusks, Carnegie Museum of Natural History Pittsburgh United States of America; 3 Southern Marine Science and Engineering Guangdong Laboratory (Zhuhai), Zhuhai 519080, China Southern Marine Science and Engineering Guangdong Laboratory (Zhuhai) Zhuhai China

**Keywords:** Asia, biodiversity, biogeography, invertebrate, morphological taxonomy

## Abstract

China is one of the most biodiverse countries worldwide, with a variety of landforms and climatic conditions. However, the diversity and biogeographic distribution of bdelloid rotifers, one of the most widespread microscopic organisms worldwide, have received little attention in China. In order to understand the species diversity of bdelloid rotifers in China, a large-scale survey of different areas (such as karst areas, islands, and plateaus) and microhabitats (such as moss, leaf litter, and soil) was conducted using 299 samples from seven provinces from 2018 to 2022. A total of 109 bdelloid rotifer species were identified, including three newly recorded genera, 26 newly recorded species, and two new species (*Adinetajinan* Wang & Yang, **sp. nov.** and *Philodinachinensis* Wang & Örstan, **sp. nov.**), increasing the species richness of bdelloid rotifers in China to 141. Morphological variations among *P.chinensis* populations from three different regions were identified. This study indicated that the species diversity of bdelloid rotifers in China is high, especially in the Oriental region, and both endemic and rare species exist in different regions and habitats. Further, the challenges are highlighted with corresponding suggestions for research on the diversity and biogeographic distribution of bdelloid rotifers in China. Notably, our findings indicated that a survey of bdelloid rotifers in China is indispensable for a comprehensive understanding of their global biodiversity and biogeography.

## ﻿Introduction

Biodiversity surveys and taxonomic studies improve our understanding of global diversity and distribution and provide up-to-date data about species adaptation to rapidly changing global environmental conditions, such as global warming and the presence of invasive species (Sarma et al. 2021). Currently, more than one-fifth of all vertebrates, invertebrates, and plants are at risk of extinction during the Anthropocene epoch (Otto 2018). Therefore, research on biodiversity is crucial for understanding the extent to which organisms are affected by human activities and formulating biodiversity conservation conventions and countermeasures (Gerber et al. 2018).

China is one of the most biodiverse regions in the world, with diverse climate zones (tropical, subtropical monsoon climate, and plateau mountain climate) and geographical areas (plateau, karst, island, estuarine plain, mountain, and hill). Its large tropical forests, in particular, host rich species diversity and a high proportion of endemic species (Zhu 2017; Mi et al. 2021). In addition, an unusually wide variety of different habitats can be found due to the abundant high mountain and plateau areas (Lei et al. 2015; Hu et al. 2020; Mi et al. 2021). Other global regions with similar climates and habitats, such as Brazil, Mexico, and India, tend to have high biodiversity and harbour many endemic species (Sarma et al. 2021). Presently, 36 biodiversity hotspots have been recognised worldwide, four of which are in Southern China, including the Mountains of Southwest China, the Himalayas, the Mountains of Central Asia, and the Indo–Burma hotspots (Mi et al. 2021). These areas provide stable refuges for animals. Notably, high levels of endemism have been reported in many Chinese organisms, including angiosperms, amphibians, and reptiles.

Bdelloid rotifers are microscopic invertebrates belonging to the phylum Rotifera that can live in different aquatic and limno–terrestrial habitats (Ricci 2017). Although bdelloid rotifers are extensively distributed across all continents because of their microscopic size and ability to enter a dormant state to tolerate extreme and adverse environmental conditions such as drought, freezing, and radiation (Ricci 2017; Shmakova et al. 2021), their diversity is not well understood compared to other microscopic organisms such as nematodes. New endemic species of bdelloid rotifers are still being discovered, particularly in remote locations and less-explored habitats. For example, Iakovenko et al. (2015) identified 12 new species in Antarctica and found that the level of endemism in bdelloids in Antarctica was higher than that on any other continents. In America, Örstan (2021) found a new genus, *Coronistomus*, that has evolved morphological structures adapted to turbulent flow habitats. In recent years, several new Asian species have been described from Korea (Song and Min 2015; Song and Lee 2020, 2022). In China, studies on bdelloid rotifers are scarce, and only sporadic surveys and a few species were documented before 2020 (Zhuge et al. 1998; Zeng et al. 2020). Currently, more than 500 species of bdelloid rotifers have been recorded globally, whereas only 113 species have been reported from China (Zeng et al. 2020; Wang et al. 2021; Wang et al. 2022, 2023). Therefore, the species diversity and geographical distribution of bdelloid rotifers in China require further investigation.

In this study, we conducted a large-scale survey of bdelloid rotifer diversity in different habitats in China, and we speculate that diverse habitats in China conceal a high level of bdelloid rotifer species diversity and that there is still great potential for the discovery of new species and opportunities for biodiversity conservation. This study aimed to (1) investigate the diversity and geographical distribution of bdelloid rotifers in different areas of China; (2) describe two new species (*Adinetajinan* sp. nov. and *Philodinachinensis* sp. nov.); and (3) provide suggestions for future study of diversity and biogeography of bdelloid rotifers.

## ﻿Materials and methods

### ﻿Study areas and sampling sites

A total of 24 sites and 299 samples from seven provinces in China were investigated from September 2018 to November 2022 (Table 1, Suppl. material 1: table S1). These sites were located in different geographical areas (plateau, karst, island, plain, and mountain) and contained different microhabitats (mosses, leaf litter, soil, lichens, aquatic plants, rivers, lakes, and streams) (Table 1). The elevation of the sampling sites varied from 0 to 4734 m. All areas differed in their topographic and climatic types, representing the major landscape types in China (Table 1).

**Table 1.** List of study sites (coordinates based on GCJ-02 reference system).

Number	Sites	Provinces	Terrain	Climate	Mean altitude	Latitude, Longitude	Number of samples
1	Linzhi	Tibet	Plateau	Plateaumountain climate	3008	29.63654°N, 94.36119°E	18
2	Bomi	Tibet	Plateau	Plateau mountain climate	2839	29.85903°N, 95.76761°E	6
3	Lasa	Tibet	Plateau	Plateau mountain climate	3660	29.65262°N, 91.13775°E	6
4	Dangxiong	Tibet	Plateau	Plateau mountain climate	4284	30.47192°N, 91.1013°E	3
5	Guilin	Guangxi	Karst	Subtropical monsoon climate	156	25.31402°N, 110.30188°E	17
6	Nanning	Guangxi	Karst	Subtropical monsoon climate	76	22.78121°N, 108.27331°E	2
7	Weizhou island	Guangxi	Island	Subtropical monsoon climate	0	21.03241131°N, 109.1202466°E	3
8	Wanshan island	Guangdong	Island	Subtropical monsoon climate	174	21.94022895°N, 113.7334365°E	4
9	Qiao island	Guangdong	Island	Subtropical monsoon climate	6	22.41488651°N, 113.6405677°E	9
10	Miaowan island	Guangdong	Island	Subtropical monsoon climate	0	21.85182962°N, 114.0113134°E	3
11	Wailingding island	Guangdong	Island	Subtropical monsoon climate	6	22.09857305°N, 114.0420408°E	3
12	Guishan island	Guangdong	Island	Subtropical monsoon climate	0	22.13308285°N, 113.8296957°E	1
13	Nanao island	Guangdong	Island	Subtropical monsoon climate	340	23.43305804°N, 117.0830221°E	9
14	Shenzhen	Guangdong	Plain	Subtropical monsoon climate	18	22.55329°N, 113.88308°E	67
15	Foshan	Guangdong	Plain	Subtropical monsoon climate	4	22.90026°N, 112.89262°E	2
16	Zhuhai	Guangdong	Plain	Subtropical monsoon climate	1	22.20907°N, 113.29673°E	9
17	Guangzhou	Guangdong	Plain	Subtropical monsoon climate	22	23.15792°N, 113.27324°E	70
18	Yongxing island	Hainan	Island	Tropical monsoon climate	0	16.82885876°N, 112.3399115°E	3
19	Haikou	Hainan	Plain	Tropical monsoon climate	10	20.02971°N, 110.32941°E	4
20	Changde	Hunan	Mountain	Subtropical monsoon climate	34	29.01871°N, 111.68072°E	49
21	Changsha	Hunan	Mountain	Subtropical monsoon climate	40	28.25591°N, 112.98626°E	3
22	Guiyang	Guizhou	Karst	Subtropical monsoon climate	1305	26.67865°N, 106.62298°E	3
23	Anshun	Guizhou	Karst	Subtropical monsoon climate	1400	26.40574°N, 106.2553°E	4
24	Chengdu	Sichuan	Mountain	Subtropical monsoon climate	479	30.65984°N, 104.10194°E	1

### ﻿Sampling and extraction of bdelloid rotifers

Terrestrial samples were collected in kraft seed bags, brought to the laboratory, and dried at room temperature. To extract bdelloid rotifers, moss, leaf litter, and lichen were soaked in deionised water for at least four hrs, shaken vigorously for 30 s, then the liquid portion was passed through a 30-µm mesh. This process was repeated three times, and the residue was rinsed in disposable petri dishes (60 mm in diameter). The sugar centrifugation method was used to extract soil rotifers (Freckman and Virginia 1993). Aquatic samples were collected and filtered through a 30-µm mesh in situ, stored in 200 ml plastic bottles, and identified in the laboratory as soon as possible. Aquatic plant samples were collected, and soaked in deionised water, and washed three times. The liquid was filtered through a 30-µm mesh. The residue was rinsed in petri dishes and examined for rotifers.

### ﻿Taxonomic and morphometric analyses

The extracts were examined under a dissecting microscope (SZX10, Olympus, Japan) and selected rotifers were transferred onto glass slides using a micropipette. Live individuals were examined under a microscope (BX51, Olympus, Japan) at a magnification range of 200–1000×. The photographs and videos of all specimens were captured for morphological measurements and analysis. The measurement steps and methods for preparing permanent slides were referred from the literature (Iakovenko et al. 2013, 2015). Species taxonomy and identification were based mainly on the keys of Donner (1965) and the website http://www.rotifera.hausdernatur.at/Species/Index. The literature on new species and morphological descriptions of published bdelloid rotifers were also used as references (Bryce 1903; Murray 1906; Milne 1916; Schulte 1954; Wang 1961; Haigh 1963; Schmid-Araya 1993; Song and Kim 2000; Iakovenko et al. 2013, 2015; Song and Min 2015; Örstan, 2018; Song and Lee 2020, 2022; Zeng et al. 2020). The biogeographic distribution of the rotifers was described according to the criteria proposed by Segers (2007). Drawings of the new species were made using Adobe Illustrator CC 2014 (Adobe Systems Inc., USA). Morphological abbreviations used in the text: **TL**, total length; **BW**, body width; **CW**, corona width; **HL**, head length; **HW**, head width; **NL**, neck length; **MxNW**, maximal neck width; **MinNW**, minimal neck width; **RaL**, ramus length; **RaW**, ramus width; **RL**, rump length; **RW**, rump width; **FL**, foot length; **FW**, foot width; **SL**, spur length; **SSW**, spur pseudosegment width (Iakovenko et al. 2013).

### ﻿Statistical analyses

The overall morphological differences of *P.chinensis* from Tibet, Sichuan, and Guizhou were tested using analysis of similarities (ANOSIM) based on all morphological parameters in Table 2 and visualised using non-metric multidimensional scaling (nMDS) in PRIMER 5.0 (Primer-E Ltd, Plymouth, UK) (Clarke and Gorley 2001). The single morphological measurements such as the total length, total width, and foot width of *P.chinensis* from the three regions were compared using One-way analysis of variance (ANOVA) followed by Tukey’s multiple comparisons test with GraphPad Prism 6.01 (GraphPad Software Inc., San Diego, CA) (Swift 1997).

**Table 2.** Morphological parameters of the new species.

	*P.chinensis* (Tibet)		*P.chinensis* (Sichuan)		*P.chinensis* (Guizhou)		*A.jinan* (Guangdong)	
	Min-Max	Mean ± SD	Min-Max	Mean ± SD	Min-Max	Mean ± SD	Min-Max	Mean ± SD
**TL**	139–246	208.545 ± 31.037	218–247	231.857 ± 11.866	157–249	220 ± 25.747	202–310	274.111 ± 32.751
**BW**	30–57	47.182 ± 7.561	52–59	55.286 ± 2.928	44–68	56.75 ± 7.3	55–72	63.222 ± 7.138
**CW**	31–48	39.667 ± 8.505	50–54	52 ± 2.828	43–48	45.8 ± 1.924	\	\
**HL**	26–39	31.333 ± 6.807	38	38 ± 0.000	28–35	31.8 ± 3.114	44–63	57.778 ± 6.360
**HW**	31–42	37 ± 5.568	43–44	43.5 ± 0.707	33–41	36.6 ± 2.966	44–52	47.111 ± 3.219
**NL**	19–26	22.8 ± 1.932	17–25	21.286 ± 2.984	17–25	21.083 ± 2.678	32–50	41.333 ± 6.062
**MxNW**	37–48	40.2 ± 3.259	39–47	42.571 ± 3.359	34–46	39.5 ± 3.503	39–48	43.889 ± 3.983
**MinNW**	25–35	29.4 ± 2.875	28–36	32.571 ± 2.699	24–32	28.667 ± 2.425	29–40	34.889 ± 3.887
**RaL**	10–17	13.4 ± 2.119	10–16	12.857 ± 2.193	10–15	13.083 ± 1.379	11–14	13.556 ± 1.014
**RaW**	6–8	6.8 ± 0.789	6–11	8 ± 1.633	6–8	6.667 ± 0.651	6–7	6.778 ± 0.441
**RL**	11–21	17.2 ± 2.741	15–23	18.143 ± 2.854	12–24	17.000 ± 3.814	29–44	39.111 ± 4.986
**RW**	17–26	22.9 ± 2.923	22–27	24.571 ± 2.225	19–28	22.833 ± 3.040	33–47	40.778 ± 5.932
**FL**	11–24	19.4 ± 4.006	9–15	13.000 ± 2.449	10–20	14.667 ± 3.916	21–33	27.778 ± 3.768
**FW**	12–16	13.9 ± 1.595	15–20	16.714 ± 1.799	11–18	14.917 ± 1.975	20–27	23.333 ± 2.550
**SL**	5–8	6.909 ± 0.944	7–10	9 ± 1	7–10	8.083 ± 1.311	\	\
**SSW**	2–4	2.909 ± 0.701	4–7	5.143 ± 1.069	2–5	3.417 ± 0.9	\	\

## ﻿Results

### ﻿Species diversity of bdelloid rotifers in China

In total, we identified 109 morphological species (including subspecies), including two new species (*Adinetajinan* sp. nov. and *Philodinachinensis* sp. nov.), three newly recorded genera (*Embata*, *Ceratotrocha*, and *Philodinavus*), and 26 newly recorded species (Table 3), Suppl. material 1: table S2). In this study, three and 22 species were newly found in the Palearctic and Oriental biogeographic regions, respectively (Segers 2007). Twenty-two species were restricted to the Palearctic or Oriental biogeographic regions, and four rare species were limited to one biogeographic region (Table 3). *Habrotrochaconstricta* (Dujardin, 1841) has the widest distribution (eight biogeographic regions). Some rare species, such as *Philodinaclypeata* Song & Lee, 2020, *Habrotrochahumilis* Schulte, 1954, *Habrotrochadiarthrantenna* De Koning, 1947, and *Macrotrachelaambigua* Donner, 1965, were identified in this study. Among the 109 bdelloid rotifers, the species number of *Macrotrachela* was the highest (31 species), followed by *Habrotrocha* (25 species) and *Philodina* (19 species).

**Table 3.** Bdelloid rotifers recorded in this study and their biogeographic distribution.

Species	Habitats	Areas	Biogeographic regions
*Adinetaacuticornis* Haigh, 1967	moss, moss on rock, leaf litter, bamboo leaf litter	Changde, Shenzhen, Yongxing island, Guilin, Nanning, Weizhou island, Linzhi	AUS, PAL^#^, ORI
*Adinetabarbata* Janson, 1893	moss on tree	Linzhi	AFR, ANT, AUS, NEA, NEO, PAL, ORI
*Adinetabeysunae* Örstan, 2018	leaf litter, bamboo leaf litter, banyan leaf litter	Changde, Shenzhen, Foshan, Guangzhou, Guiyang, Anshun, Guilin, Nanning, Haikou	NEA, ORI
*Adinetacuneata* Milne, 1916	moss, moss on rock, moss on tree, moss on soil, leaf litter, banyan leaf litter, bamboo leaf litter	Changde, Guangzhou, Foshan, Zhuhai, Shenzhen, Anshun, Guilin	AFR, AUS, NEA, PAL, ORI
*Adinetajinan* sp. nov.	moss, leaf litter	Shenzhen	ORI^#^
*Adinetaoculata* (Milne, 1886)	leaf litter	Shenzhen	NEO, PAL, ORI
*Adinetaricciae* Segers & Shiel, 2005	aquatic plant in river	Changde	AUS, PAL, ORI
*Adinetasteineri* Bartoš, 1951	leaf litter	Guangzhou	ANT, AUS, NEA, NEO, PAL, ORI
*Adinetavaga* (Davis, 1873)	moss, moss on rock, leaf litter, bamboo leaf litter,banyan leaf litter, river	Changde, Foshan, Shenzhen, Yongxing island, Wanshan island, Guiyang, Anshun, Guilin, Nanning, Weizhou island, Linzhi, Lasa, Bomi, Dangxiong, Haikou	AFR, ANT, AUS, NEA, NEO, ORI, PAL
*Adinetavagaminor* Bryce, 1893	leaf litter	Changde, Guangzhou	AUS, NEA, ORI, PAL
*Bradyscelaclauda* (Bryce, 1893)	leaf litter, bamboo leaf litter	Changde, Guangzhou, Foshan, Shenzhen, Anshun	AUS, NEO, PAL, ORI
*Bradyscelahoonsooi* Song & Min, 2015	moss, leaf litter	Shenzhen	PAL, ORI
*Habrotrochaangusticollis* (Murray, 1905)	moss, moss on tree, leaf litter	Shenzhen	AFR, ANT, AUS, NEA, NEO, ORI, PAL
*Habrotrochabidens* (Gosse, 1851)	moss, moss on soil, moss on tree, moss on rock, leaf litter,banyan leaf litter, bamboo leaf litter, soil, aquatic plant in river	Changde, Guangzhou, Shenzhen, Foshan, Zhuhai, Guiyang, Guilin, Weizhou island, Lasa, Linzhi, Haikou	AFR, AUS, NEA, NEO, ORI, PAL
*Habrotrochaconstricta* (Dujardin, 1841)	moss, moss on tree, leaf litter, bamboo leaf litter, banyan leaf litter, soil	Changde, Guangzhou, Foshan, Shenzhen, Guiyang, Lasa, Linzhi	AFR, ANT, AUS, NEA, NEO, PAC, PAL, ORI
*Habrotrochadiarthrantenna* De Koning, 1947	leaf litter	Changde	PAL, ORI
*Habrotrochaelusa* Milne, 1916*	bamboo leaf litter	Lasa	AFR, ANT, AUS, PAL
*Habrotrochaflava* Bryce, 1915	moss, moss on tree, moss on rock, leaf litter, bamboo leaf litter, soil	Changde, Guangzhou, Zhuhai, Shenzhen, Yongxing island, Qiao island, Anshun, Guilin, Linzhi	AUS, NEA, PAL, ORI
*Habrotrochafilum* Donner, 1949*	leaf litter	Shenzhen	PAL, ORI^#^
*Habrotrochagracilis* Montet, 1915*	moss	Shenzhen	AUS, PAL, ORI^#^
*Habrotrochahumilis* Schulte, 1954	moss	Changde	PAL, ORI
*Habrotrochaligula* Bryce, 1913	moss, leaf litter, banyan leaf litter, soil, *Leontopodiumleontopodioides*	Foshan, Shenzhen, Bomi, Linzhi, Dangxiong	AUS, NEA, PAL, ORI
*Habrotrochaligulaligula* Bryce, 1913	leaf litter, banyan leaf litter	Changde, Foshan, Miaowan island	AUS, NEA, PAL, ORI
*Habrotrochalata* (Bryce, 1892)*	soil	Shenzhen	AFR, AUS, NEA, NEO, ORI, PAL
*Habrotrochanodosa* (Murray, 1906)*	leaf litter, bamboo leaf litter	Miaowan island, Shenzhen, Anshun	AFR, NEO, ORI, PAC, PAL
*Habrotrochapavida* Bryce, 1915*	moss, bamboo leaf litter	Changde, Shenzhen	NEA, PAL, ORI^#^
*Habrotrochaparvipes* Donner, 1951	leaf litter	Changde	PAL, ORI
*Habrotrochaquinquedensdoornensis* De Koning, 1947*	*Leontopodiumleontopodioides*	Dangxiong	PAL
*Habrotrocharosa* Donner, 1949	moss on rock, leaf litter, bamboo leaf litter	Changde, Shenzhen, Guiyang, Weizhou island, Dangxiong, Lasa	AFR, AUS, NEA, NEO, PAL, ORI
*Habrotrocharara* Donner, 1949	moss	Changde	PAL, ORI
*Habrotrochascepanotrochoides* De Koning, 1947*	leaf litter	Guangzhou, Shenzhen	AUS, PAL, ORI^#^
*Habrotrochastenostephana* Schulte, 1954*	moss, leaf litter	Shenzhen	AFR, PAL, ORI^#^
*Habrotrochathienemanni* Hauer, 1924	bamboo leaf litter	Changde	PAL, ORI^#^
*Habrotrochathienemannirubella* Donner, 1951*	leaf litter	Miaowan island	PAL, ORI^#^
*Habrotrochatranquilla* Milne, 1916	leaf litter	Changde	AFR, AUS, PAL, ORI
*Habrotrochatripus* (Murray, 1907)*	moss on rock, leaf litter, soil	Wanshan island, Shenzhen, Nanning, Linzhi	AUS, NEO, PAL, ORI^#^
*Habrotrochavisa* Donner, 1954*	moss, moss on soil	Shenzhen	PAL, ORI^#^
*Otostephanosauriculatus* (Murray, 1911)*	leaf litter	Shenzhen	AFR, AUS, NEA, PAC, PAL, ORI^#^
*Otostephanosauriculatusbilobatus* Hauer, 1939*	leaf litter	Guangzhou	AUS, PAL, ORI^#^
*Otostephanosdonneri* Bartoš, 1959	moss, leaf litter, soil	Changde, Guangzhou, Shenzhen	AUS, PAL, ORI
*Otostephanosregalis* Milne, 1916	moss, moss on rock, moss on tree, leaf litter	Guangzhou, Shenzhen, Guilin, Linzhi	AFR, PAL, ORI
*Otostephanostorquatus* (Bryce, 1913)	moss on rock	Nanning	AFR, AUS, NEA, PAL, ORI
*Otostephanostorquatustorquatus* (Bryce, 1913)	moss, moss on soil, moss on rock, leaf litter, bamboo leaf litter	Changde, Shenzhen, Guilin	AUS, NEA, PAL, ORI
*Otostephanostorquatusamoenus* Milne, 1916	moss, moss on soil, moss on rock, leaf litter, bamboo leaf litter	Changde, Guilin, Bomi	AFR, PAL, ORI
*Scepanotrochasemitecta* Donner, 1951	leaf litter	Changde	PAL, ORI
*Rotariacitrina* (Ehrenberg, 1838)	aquatic plant in pond	Shenzhen	AFR, AUS, NEA, PAL, ORI
*Rotariamacroceros* (Gosse, 1851)	aquatic plant in pond	Shenzhen	AFR, AUS, NEA, NEO, ORI, PAL
*Rotariamontana* (Murray, 1911)*	moss, moss on tree	Linzhi, Bomi, Lasa	AUS, ORI, PAL^#^
*Rotariarotatoria* (Pallas, 1766)	aquatic plant in pond, river, lake	Changde, Guangzhou, Shenzhen	AFR, AUS, NEA, NEO, ORI, PAC, PAL
*Rotariasordida* (Western, 1893)	moss, moss on tree, moss on soil, moss on rock, leaf litter, bamboo leaf litter, soil, lichen	Changde, Changsha, Zhuhai, Shenzhen, Yongxing island, Wanshan island, Wailingding island, Qiao island, Anshun, Guilin, Weizhou island, Linzhi, Bomi, Haikou	AFR, AUS, NEA, NEO, ORI, PAC, PAL
*Rotariatardigrada* (Ehrenberg, 1830)	moss, aquatic plant in lake, aquatic plant in pond, lake	Changde, Guangzhou, Shenzhen	AFR, AUS, NEA, NEO, ORI, PAL
*Rotariatridens* (Montet, 1915)	aquatic plant in pond	Shenzhen	AUS, NEA, NEO, PAL, ORI
*Dissotrochaaculeata* (Ehrenberg, 1830)	aquatic plant in pond	Shenzhen	AFR, AUS, NEA, NEO, ORI, PAL
*Dissotrochamacrostyla* (Ehrenberg, 1838)	river,stream	Changde, Nanao island	AFR, AUS, NEA, NEO, ORI, PAL
*Pleuretrabrycei* (Weber, 1898)	moss, moss near stream	Nanao island, Shenzhen	AFR, AUS, NEA, NEO, PAL, ORI
*Embatahamata* (Murray, 1906)*	stream	Nanao island	AUS, PAL, ORI^#^
*Macrotrachelaaculeata* Milne, 1886	moss, leaf litter, bamboo leaf litter	Changde, Shenzhen, Guiyang, Anshun, Guilin, Nanning	AFR, AUS, NEA, NEO, PAL, ORI
*Macrotrachelaambigua* Donner, 1965	leaf litter, bamboo leaf litter	Changde, Shenzhen	PAL, ORI
*Macrotrachelabrevilabris* De Koning, 1947*	moss, moss on rock, bamboo leaf litter	Shenzhen, Guilin, Bomi, Dangxiong	AUS, PAL, ORI^#^
*Macrotrachelaconcinna* (Bryce, 1912)*	leaf litter, bamboo leaf litter	Qiao island, Guilin	ANT, AUS, NEO, PAL, ORI^#^
*Macrotracheladecora* (Bryce, 1912)*	moss, soil	Shenzhen	AUS, NEA, PAL, ORI^#^
*Macrotrachelaehrenbergii* (Janson, 1893)	moss, moss on tree, leaf litter, bamboo leaf litter	Changde, Changsha, Guangzhou, Shenzhen, Linzhi, Bomi	AFR, AUS, NEA, NEO, ORI, PAC, PAL
*Macrotrachelaformosa* (Murray, 1906)*	moss on rock, bamboo leaf litter	Wailingding island, Linzhi	AFR, AUS, NEO, ORI, PAL
*Macrotrachelahabita* (Bryce, 1894)	moss, moss on rock, moss on soil, moss on tree, leaf litter, bamboo leaf litter, soil	Changde, Guangzhou, Zhuhai, Nanao island, Shenzhen, Yongxing island, Wanshan island, Anshun, Guilin, Nanning, Linzhi, Haikou	AFR, ANT, AUS, NEA, NEO, ORI, PAL
*Macrotrachelainermis* Donner, 1965	leaf litter, bamboo leaf litter	Changde, Shenzhen	PAL, ORI
*Macrotrachelainsolita* De Koning, 1947	moss, moss on rock, bamboo leaf litter	Changde, Zhuhai, Wanshan island, Linzhi	ANT, AUS, NEA, NEO, PAL, ORI
*Macrotrachelainduta* Donner, 1951	moss, leaf litter, bamboo leaf litter, aquatic plant in river	Changde, Yongxing island, Miaowan island, Shenzhen, Lasa	PAL, ORI
*Macrotrachelakallosoma* (Schulte, 1954)	moss	Changde	ANT, AUS, NEO, PAL, ORI
*Macrotrachelalibera* Donner, 1949	leaf litter	Changde	PAL, ORI
*Macrotrachelamultispinosa* Thompson, 1892	moss, moss on tree, moss on rock, leaf litter, bamboo leaf litter, soil	Changde, Zhuhai, Wailingding island, Qiao island, Shenzhen, Guiyang, Bomi	AFR, AUS, NEA, NEO, ORI, PAL
*Macrotrachelamultispinosamultispinosa* Thompson, 1892	moss, moss on tree, moss on rock, leaf litter, bamboo leaf litter	Changde, Changsha, Anshun, Guilin, Linzhi	AFR, AUS, NEA, NEO, ORI, PAL
*Macrotrachelamultispinosabrevispinosa* (Murray, 1908)	moss, moss on tree, moss on soil, moss on rock, leaf litter, bamboo leaf litter,soil	Changde, Changsha, Shenzhen, Yongxing island, Wanshan island, Guilin, Nanning, Weizhou island, Linzhi, Haikou	AFR, AUS, NEO, ORI, PAL
*Macrotrachelamultispinosaflagellata* Bartoš, 1951	moss, moss on tree, leaf litter	Changde, Shenzhen	ORI
*Macrotrachelanixa* Donner, 1962*	moss	Guilin	ANT, NEO, PAL, ORI^#^
*Macrotrachelanana* (Bryce, 1912)	moss, leaf litter	Changde, Shenzhen	AFR, AUS, NEA, NEO, PAL, ORI
*Macrotrachelapacifica* (Murray, 1911)*	soil	Shenzhen	PAC, ORI^#^
*Macrotrachelapinnigera* (Murray, 1908)*	moss, leaf litter	Shenzhen, Guilin	AFR, ORI^#^
*Macrotrachelapapillosa* Thompson, 1892	leaf litter, bamboo leaf litter, banyan leaf litter	Changde, Foshan	AFR, AUS, NEA, NEO, ORI, PAL
*Macrotrachelaplicataplicata* (Bryce, 1892)	leaf litter	Changde	AFR, AUS, NEA, PAL, ORI
*Macrotrachelaquadricornifera* Milne, 1886	moss, moss on rock, leaf litter, bamboo leaf litter, soil	Changde, Guangzhou, Shenzhen, Zhuhai, Yongxing island, Miaowan island, Wanshan island, Wailingding island, Qiao island, Anshun, Guilin, Nanning, Weizhou island, Linzhi, Bomi, Haikou	AFR, ANT, AUS, NEA, NEO, ORI, PAL
*Macrotrachelaquadricorniferarigida* Milne, 1916	leaf litter	Changde, Miaowan island	AFR, AUS, NEO, PAL, ORI
*Macrotrachelaquadricorniferaloricata* Donner, 1965	moss, leaf litter	Changde, Yongxing island	AFR, AUS, NEA, NEO, PAL, ORI
*Macrotrachelaquadricorniferavanoyei* Schepens,1954	moss	Changde	ORI
*Macrotrachelaquadricorniferaligulata* Bērziņš, 1950*	bamboo leaf litter	Guiyang	PAL, ORI^#^
*Macrotrachelatimida* Milne, 1916	moss, moss on rock, moss on soil, moss on tree, leaf litter, bamboo leaf litter	Changde, Shenzhen, Yongxing island, Guilin, Bomi, Linzhi, Haikou	AFR, AUS, NEO, PAL, ORI
*Macrotrachelatimidainquies* Milne, 1916	leaf litter	Changde	AFR, NEO, PAL, ORI
*Macrotrachelatimidatimida* Milne, 1916	moss, leaf litter	Changde, Guangzhou	AFR, AUS, PAL, ORI
*Philodinaacuticornis* Murray, 1902	moss, moss on rock, moss on tree, leaf litter	Wanshan island, Wailingding island, Shenzhen, Guilin	AFR, AUS, NEA, NEO, PAL, ORI
*Philodinacitrina* Ehrenberg, 1830	moss, moss on rock, river, aquatic plant in pond	Changde, Shenzhen, Weizhou island	AFR, AUS, NEA, NEO, ORI, PAL
*Philodinaclypeata* Song & Lee, 2020	moss, leaf litter, soil	Changde, Guangzhou, Shenzhen	PAL, ORI
*Philodinachildi* Milne, 1916	leaf litter	Changde	AFR, ORI
*Philodinachinensis* sp. nov.	moss, moss on tree, bamboo leaf litter	Anshun, Chengdu, Lasa, Bomi	PAL^#^, ORI^#^
*Philodinagrandis* Milne, 1916	leaf litter	Changde, Guangzhou, Shenzhen	AFR, AUS, PAL, ORI
*Philodinaindica* Murray, 1906	moss	Shenzhen, Guilin	NEA, PAL, ORI
*Philodinamegalotrocha* Ehrenberg, 1832	aquatic plant in pond, river	Changde, Shenzhen	AFR, AUS, NEA, NEO, ORI, PAL
*Philodinanemoralis* Bryce, 1903	moss	Changde, Linzhi	AFR, AUS, NEA, PAL, ORI
*Philodinanitida* Milne, 1916	moss	Guilin	AFR, AUS, ORI
*Philodinaplena* (Bryce, 1894)	moss, moss on rock, moss on tree, leaf litter, bamboo leaf litter, banyan leaf litter, aquatic plant in river	Changde, Foshan, Guishan island, Shenzhen, Anshun, Guilin, Nanning, Weizhou island, Linzhi, Lasa, Bomi	AFR, ANT, AUS, NEA, NEO, PAL, ORI
*Philodinaproterva* Milne, 1916	moss, moss on rock, leaf litter, banyan leaf litter	Changde, Foshan, Shenzhen, Guilin, Lasa	AFR, AUS, NEA, PAL, ORI
*Philodinaparvicalcar* De Koning, 1947	moss, leaf litter	Bomi, Linzhi	PAL, ORI
*Philodinarapida* Milne, 1916	moss, leaf litter, bamboo leaf litter	Changde, Guangzhou, Zhuhai, Shenzhen, Anshun	AFR, NEO, PAL, ORI
*Philodinarugosa* Bryce, 1903	moss, moss on tree, moss on rock, bamboo leaf litter	Changde, Changsha, Shenzhen, Wanshan island, Qiao island, Guilin, Haikou	AFR, AUS, NEA, NEO, PAL, ORI
*Philodinaroseola* Ehrenberg, 1832	leaf litter, bamboo leaf litter	Shenzhen, Linzhi	AFR, AUS, NEA, NEO, PAL, ORI
*Philodinascabra* Milne, 1916	moss	Guilin	AFR, PAL, ORI
*Philodinatranquilla* Wulfert, 1942	moss, leaf litter, banyan leaf litter	Changde, Shenzhen	AUS, PAL, ORI
*Philodinavorax* (Janson, 1893)	moss, moss on tree, moss on soil, moss on rock, leaf litter, bamboo leaf litter, soil	Changde, Changsha, Shenzhen, Guishan island, Wanshan island, Anshun, Nanning, Linzhi, Bomi, Lasa	AFR, AUS, NEA, NEO, ORI, PAL
*Mniobiamagna* (Plate, 1889)	moss, leaf litter	Changde, Wailingding island, Anshun, Lasa	AUS, NEA, NEO, PAL, ORI
*Ceratotrochacornigera* (Bryce, 1893)*	moss on rock, moss on tree	Dangxiong, Linzhi	ANT, AUS, NEA, NEO, PAL
*Philodinavusparadoxus* (Murray, 1905)*	stream, water inlet of pool	Guangzhou, Nanao island	AUS, NEA, PAL, ORI^#^

*: New records of bdelloid rotifers from China. #: New records of bdelloid rotifers from specific biogeographic region.

In the investigated study areas, the most widespread species was *Macrotrachelaquadricornifera* Milne, 1886, which was found in 16 sites, followed by *Adinetavaga* (Davis, 1873) (15 sites), *Rotariasordida* (Western, 1893) (14 sites), and *Macrotrachelahabita* (Bryce, 1894) (12 sites). Further, 42 species were distributed at only one site. Therefore, these may be rare species in China. Among the 24 sites, the highest species number of bdelloid rotifers was observed in the Shenzhen area (64 species), followed by Changde (63 species) and Guilin (27 species) (Table 3).

Most bdelloid rotifer species were collected from leaf litter habitat (77 species), followed by moss (66 species), and soil (16 species) habitats. In addition, we found 16 aquatic bdelloid rotifers that live in various aquatic environments, such as rivers, lakes, and ponds, and can also attach to aquatic plant surfaces (Table 3). Many bdelloid rotifer species can coexist in two or more habitats, such as *R.sordida* and *Macrotrachelamultispinosa* Thompson, 1892, which have strong environmental adaptation abilities. Other limno–terrestrial species, such as *Macrotrachelainduta* Donner, 1951, have also been accidentally found in aquatic environments. Similarly, some aquatic species occasionally exist in limno–terrestrial environments, such as *Rotariatardigrada* (Ehrenberg, 1830), which is found in mosses near ponds. The species number of bdelloid rotifers observed in different habitats was different. Among the limno–terrestrial habitats, leaf litter had the highest species number, followed by moss, soil, and lichen. The highest number of bdelloid species in aquatic habitats was found in aquatic plants (12 species), and the lowest was found in lakes (2 species). In other specialised habitats, such as *Leontopodiumleontopodioides* growing in the Tibetan Plateau, only two bdelloid rotifer species (*Habrotrochaligula* Bryce, 1913 and *Habrotrochaquinquedensdoornensis* De Koning, 1947) were found.

**Figure 1.**
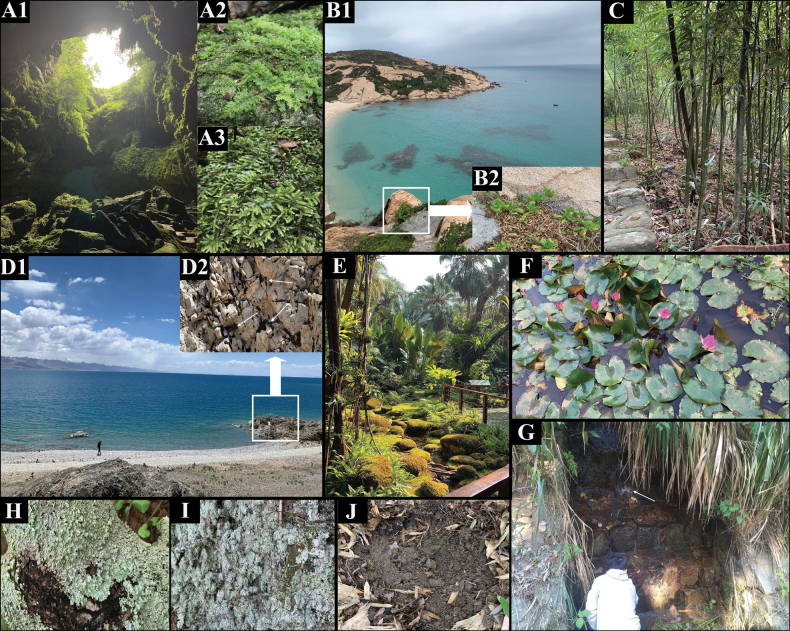
Different habitats investigated in this study **A1** Qianlong doline, Guilin, Guangxi **A2, A3** Moss on rock in Qianlong doline **B** Miaowan island, Guangdong **B2** Shrubs leaf litter in Miaowan island **C** Bamboo leaf litter in Fairy Lake Botanical Garden, Shenzhen, Guangdong **D1** Namucuo lake (4711 m a.s.l.), Dangxiong, Tibet **D2** Moss on rock near Namucuo lake. The white arrows show black moss growing in the crevices **E** Moss land near a brook in Fairy Lake Botanical Garden, Shenzhen, Guangdong **F** Lotus pond in Fairy Lake Botanical Garden, Shenzhen, Guangdong **G** Stream in Nanao island, Guangdong. The white arrow indicates stream flowing down from the mountain **H** Lichen on tree, Linzhi, Tibet **I** Lichen on soil, Guangdong **J** Soil, Shenzhen, Guangdong.

**Figure 2.**
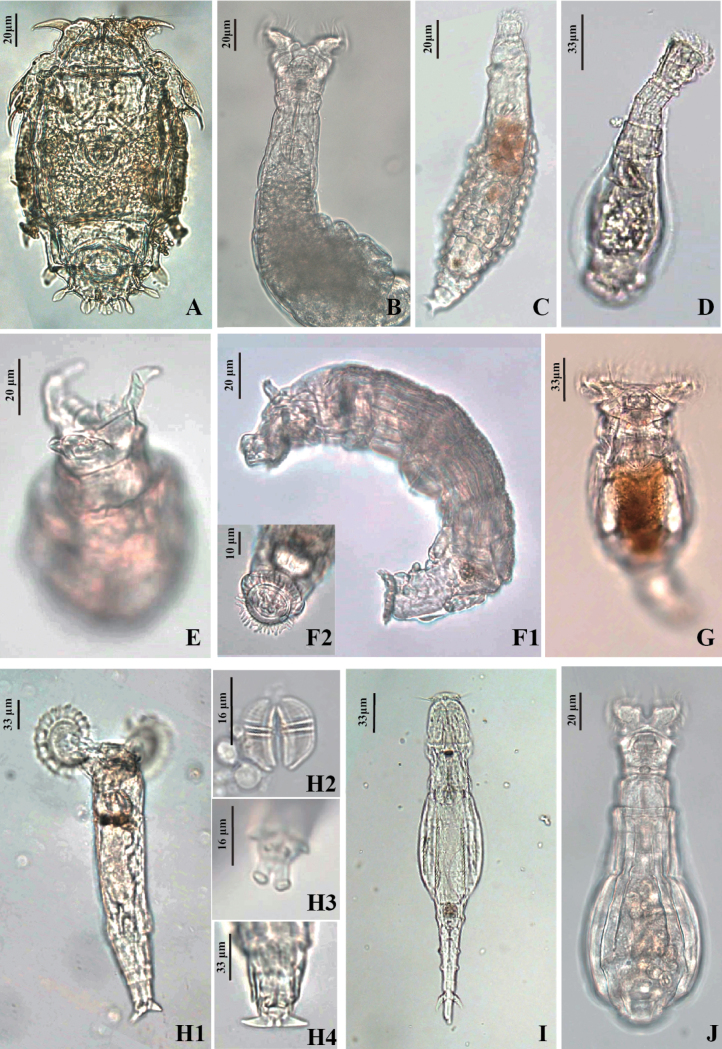
Some of the bdelloid species found in China **A***Macrotrachelapinnigera***B***Macrotrachelanixa***C***Macrotrachelaformosa***D***Habrotrochaangusticollis***E***Ceratotrochacornigera***F1***Bradyscelahoonsooi***F2** toes and papillae **G***Philodinamegalotrocha***H1***Embatahamata***H2** trophi **H3** toes **H4** spur **I***Adinetabeysunae***J***Habrotrochapavida*.

Among the different geographical regions investigated in China, plain areas had the highest bdelloid rotifer species number (72 species), followed by mountain areas (64 species), karst areas (39 species), plateau areas (35 species), and islands (31 species). Notably, these regions have different topographic and climatic characteristics (Fig. 1); and some rare species with special morphological characteristics are newly found in these regions (Fig. 2).

### ﻿Different geographical areas, habitats, and newly recorded species in China

Different geographical areas and diverse habitats in China were investigated, and many rare and unique bdelloid species were identified (Fig. 1). Study areas and habitats included karst, islands, plateaus, mosses, leaf litter, lichens, soil, aquatic plants, rivers, lakes, and streams.

#### ﻿Karst

Karst landforms are common in Southwest China’s Guangxi and Guizhou provinces, making it one of the largest karst regions in the world.

##### Representative location

Qianlong Doline, Guilin, Guangxi Province, China. The Qianlong Doline is a typical karst landscape with an underground river and a wide variety of plants (Fig. 1A). The temperature inside the doline was low, and the humidity was high. Many different types of mosses with high moisture content were found on the rocks inside the doline (Fig. 1A2, A3).

##### Newly recorded species

*Macrotrachelapinnigera* (Murray, 1908) and *Macrotrachelanixa* Donner, 1962 (Fig. 2A, B).

#### ﻿Islands

Eight islands in Southern China were investigated in this study, of which the island furthest from the mainland was Yongxing Island (339 km as the crow flies from Sanya, Hainan), and the largest island was Nanao Island (118 km^2^), and the smallest one was Miaowan Island (1.4 km^2^).

##### Representative locations

Nanao Island, Shantou, Guangdong Province; Miaowan Island, Zhuhai, Guangdong Province. Compared to Nanao Island, which is rich in forest resources, Miaowan Island is farther away from the mainland, and the plant on the island is mainly shrubs owing to the strong sea breeze (Fig. 1B).

##### Newly recorded species

We found *Embatahamata* (Murray, 1906) (Fig. 2H) in a stream running down a mountain in Nanao Island (white arrow in Fig. 1G). Further, we found *M.formosa* (Murray, 1906) in the shrub leaf litter on Miaowan Island (Fig. 2C).

#### ﻿Plateau

In this study, we investigated four sites on the Tibetan Plateau with average elevations ranging from 2323 to 4734 m.

##### Representative location

Lake Namucuo, Dangxiong, Tibet. Namucuo Lake is a large plateau lake with an altitude of 4711 m (Fig. 1D1), and we collected many small black mosses from the rock crevices adjacent to the lake (Fig. 1D2).

##### Newly recorded species

*Ceratotrochacornigera* (Bryce, 1893), with long horn-shaped appendages on either side of the crown, was found in this moss habitat (Fig. 2E).

#### ﻿Leaf litter, moss, and aquatic plants

In this study, we conducted a large number of surveys on leaf litter habitats, among which bamboo leaf litter was the most intensively studied because of its wide distribution and abundance in China. In addition, mosses, lichens (Fig. 1H, I), soil (Fig. 1J), and aquatic plants were the most studied habitats for bdelloid rotifers.

##### Representative location

Fairy Lake Botanical Garden, Shenzhen, Guangdong Province.

##### Representative species

*Adinetabeysunae* and *Habrotrochaangusticollis* (Murray, 1905) were found in the leaf litter of the bamboo forests (Fig. 1C). In the moss-covered area of the Fairy Lake Botanical Garden (Fig. 1E), we found *Bradyscelahoonsooi* Song & Min, 2015 (Fig. 2F) and *Habrotrochapavida* Bryce, 1915 (Fig. 2J). Similarly, we found *Philodinamegalotrocha* Ehrenberg, 1832 (Fig. 2G) attached to the stems and leaves of lotus in large quantities (Fig. 1G).

### ﻿Descriptions of new species

**Figure 3.**
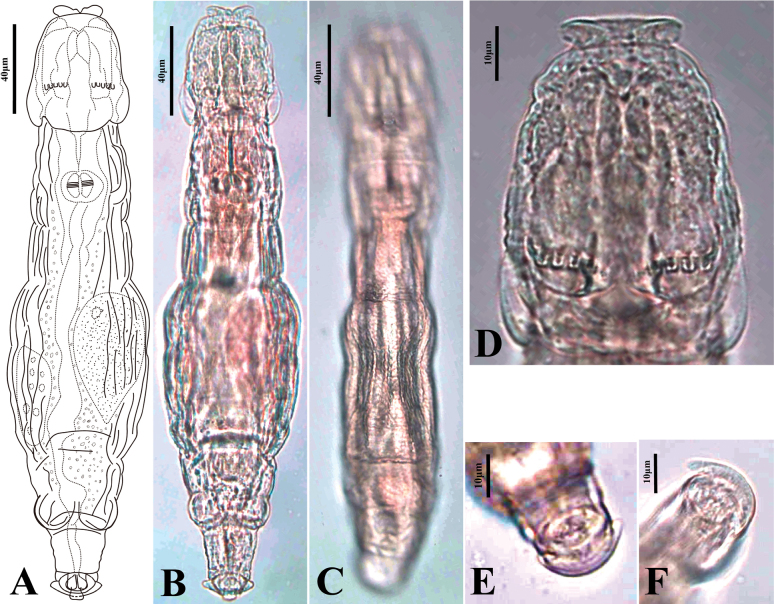
*Adinetajinan* sp. nov. **A** creeping, ventral view **B** creeping, ventral view **C** creeping, dorsal view **D** head, ventral view **E** spurs, ventral view **F** spurs, ventral view.

**Figure 4.**
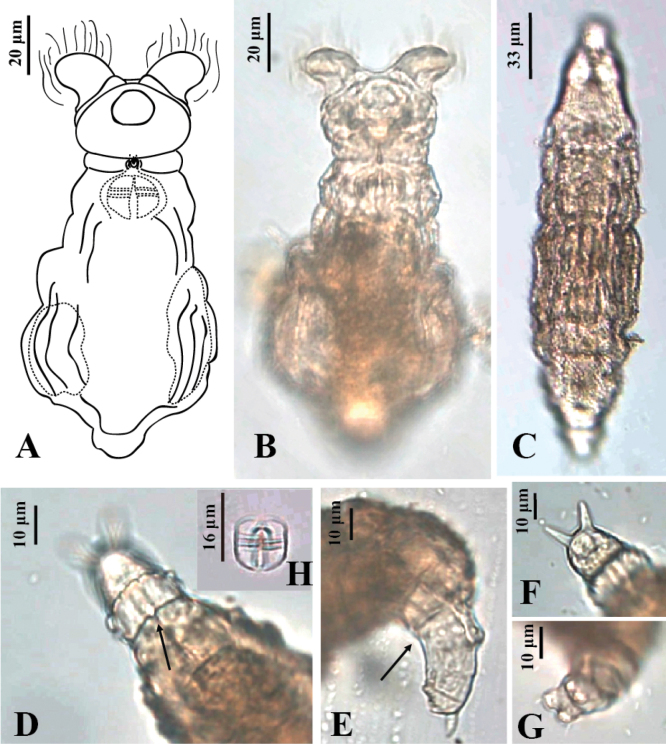
*Philodinachinensis* sp. nov. **A** feeding, dorsal view **B** paratype, feeding, dorsal view **C** holotype, creeping, dorsal view **D** holotype, foot, dorsal view **E** holotype, foot, lateral view **F** holotype, spur, dorsal view **G** holotype, toes, ventral view **H** trophi.

Here, we provide descriptions and illustrations of two new species, *Adinetajinan* sp. nov. (Fig. 3) and *Philodinachinensis* sp. nov. (Fig. 4)

#### ﻿Family Adinetidae Hudson & Gosse, 1889

**Genus *Adineta* Hudson & Gosse, 1886**

##### ﻿ Adineta jinan

Taxon classificationAnimaliaAdinetidaAdinetidae

Wang & Yang sp. nov.

D55BC0AD-BD38-571A-8E22-E81B8199BE19

https://zoobank.org/57A69315-DA3D-447C-93DF-39D19090D7BA

###### Material examined.

***Holotype***: China • one female on permanent slide; Guangdong province, Shenzhen city, Shenzhen Fairy Lake Botanical Garden; 22.5786°N, 114.1797°E; 77 m a.s.l.; collected on 5 Dec. 2021; W.B. Wang leg.; collected from leaf litter and mosses near a streamlet; SYSBM Ro-20221229-04. ***Paratypes***: • two females on permanent slides; same data as for holotype; SYSBM Ro-20221229-05 and SYSBM Ro-20221229-06. All deposited in the Museum of Biology, Sun Yatsen University, Guangzhou, China.

###### Differential diagnosis.

Spurs short stubs at right angles to foot and united by a narrow ridge. A large spherical swelling present on each side of anal pseudosegment. Integument rough, has a sculpture of delicate granulation.

###### Description.

Body of medium size, not wide, flattened. Integument transparent, rough. Interior of trunk always coloured mahogany, head and foot lighter in colour. Head trapezoid, wider in posterior part; HL 19.9–22.9% of TL, HW 69.8-100% of HL. Distal rostral lamella wide and flat, with a V-shaped notch in the middle; without sensory bristles. Five U-shaped gaps on each rake. Neck of moderate length and width, significantly narrower than posterior head; NL 12.3–16.6% of TL. Trunk vase-shaped; BW 18.8–29.2% of TL. A large spherical swelling present on each side of anal pseudosegment; RL 13.4–14.9% of TL, RW 84.6–127% of RL. Wide foot of three pseudosegments, short; FL 8.8–12.3% of TL, FW 64.5–104.3% of FL. Spurs short stubs at right angles to foot and united by a narrow ridge. Three stubby toes. No eyespots. Trophi, 11–14 μm long and 6–7 μm wide; dental formula 2/2 (Fig. 3).

###### Comparison with other species.

There is no other known *Adineta* species with spurs similar to those of *Adinetajinan* sp. nov. *Adinetacuneata* Milne, 1916 also has very short spurs that are widely separated, but not joined across the foot. In addition, there is a deep constriction between the pseudosegment carrying the spurs and the one before it (Milne 1916), which is absent in *Adinetajinan* sp. nov. Schulte (1954: Abb. 40) and Haigh (1963: fig. 1A) gave brief descriptions of an *Adineta* variety with short spurs that were apparently curved anteriorly and which they both identified tentatively as *A.cuneata*. However, the rotifer they observed did not match the description of *A.cuneata* and may have been *Adinetajinan* sp. nov. or a variety similar to it.

###### Measurements.

See Table 2.

###### Distribution.

The new species is known only from Shenzhen city.

###### Etymology.

The species is named after Jinan University, where the authors WW, Y Y, and QW work.

#### ﻿Family Philodinidae Ehrenberg, 1838

**Genus *Philodina* Ehrenberg, 1830**

##### ﻿ Philodina chinensis

Taxon classificationAnimaliaPhilodinidaPhilodinidae

Wang & Örstan sp. nov.

2221CC50-BC4C-5BE2-B0AA-CF2575F75EAB

https://zoobank.org/E59046CF-38B1-4C73-BC10-B41A61397FFC

###### Material examined.

***Holotype***: China • one female on permanent slide; Sichuan province, Chengdu city, Chengdu zoo; 30.7104°N, 104.1063°E; 505 m a.s.l.; collected on 6 Jul. 2019; W.B. Wang leg.; collected from leaf litter; SYSBM Ro-20221229-01. ***Paratypes***: • two females on permanent slides; same data as for holotype; SYSBM Ro-20221229-02 and SYSBM Ro-20221229-03. All deposited in the Museum of Biology, Sun Yatsen University, Guangzhou, China.

###### Differential diagnosis.

Dorsal posterior border of anal pseudosegment with wavy transverse ridge of multiple knobs; posterior border of pre-anal pseudosegment with V-shaped notch.

###### Description.

Body of medium size. Rostrum of moderate length; lamella with two tiny lobes. Trochal discs wider than head but not exceeding widest part of trunk when feeding. CW 113.6–125.6% of HW, HL 15.4–17.4% of TL. Upper lip medium height, with two distinctively separated wide lobes. Sulcus wide, without ligula. Neck stout, moderately long. Antenna long, approximately 1/2 of bearing pseudosegment width. Trunk stout; integument of trunk rough, sticky, with bark-like longitudinal ridges, covered with small papilla; BW 21.8–25.7% of TL. V-shaped notch on posterior border of pre-anal pseudosegment. Wavy transverse ridge consisting of multiple knobs on posterior border of the anal pseudosegment, no knobs on ventral side. RL/TL 6.3–9.3%, RW/RL 117.4–153.3%. Foot short, of four pseudosegments; FL 3.8–6.9% of TL; FW 100–200% of FL. Spurs of moderate length, peg-shaped, divergent; interspace width slightly wider than spur width; SL 142.9–250% of SSW. Four thick toes. No eyespots. Trophi, 10–16 μm long and 6–11 μm wide; dental formula 2+1/1+2. Egg oval and surface smooth, with protrusion on one side (Fig. 4).

###### Comparison with other species.

Similar to subspecies *P.rugosacoriacea* Bryce, 1903 in the prominent ridges of the skin-folds of the trunk and shagreened integument. However, *P.rugosacoriacea* has a straight transverse ridge on the first foot pseudosegment (Bryce 1903; Song and Kim 2000); in comparison, the transverse ridge in *P.chinensis* sp. nov. consists of knobs. In addition, *P.rugosa* has eyespots (Bryce 1903; Song and Kim 2000) that are absent in *P.chinensis* sp. nov. The dental formula of *P.rugosa* is 3/3 (Bryce 1903), but that of *P.chinensis* sp. nov. is 2+1/1+2.

###### Measurements.

See Table 2. The proportion data of morphological parameters in the description were obtained from the Sichuan specimens of *P.chinensis*.

###### Distribution.

The new species is widely distributed in southern China. Seven specimens were documented in Chengdu city, Sichuan province. Thirteen specimens were documented in Guiyang city, Guizhou province. Thirteen specimens were documented in Lasa city, Tibet province.

###### Etymology.

The new species is named after the country of China, where it is widely distributed.

### ﻿Morphological analysis of *Philodinachinensis* sp. nov.

**Figure 5.**
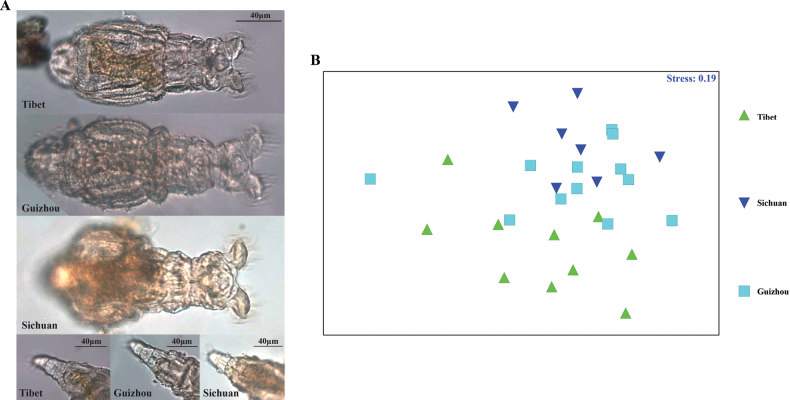
*Philodinachinensis***A** light microscopy photographs of specimens from Tibet, Guizhou, and Sichuan **B**nMDS plot of overall morphological characteristic parameters of *P.chinensis* from Tibet, Guizhou, and Sichuan.

The ANOSIM analysis revealed significant overall morphological parameters differences among *P.chinensis* from different regions (ANOSIM, global R = 0.274; *p* < 0.01) (Fig. 5B). *Philodinachinensis* from Tibet showed significant overall morphological differences from *P.chinensis* from Sichuan (ANOSIM, R = 0.373; *p* < 0.01) and Guizhou (ANOSIM, R = 0.327; *p* < 0.01); however, the overall differences between Guizhou and Sichuan species were not significant (ANOSIM, *p >* 0.05). The comparison of single morphological parameters showed that the total length of the *P.chinensis* was similar in three regions (One-way ANOVA, *p* > 0.05). But the body width of Tibetan specimens was significantly narrower than that of Sichuan (One-way ANOVA, *p* < 0.05) and Guizhou species (One-way ANOVA, *p* < 0.01), and the foot width of Tibetan specimens was significantly shorter than that of Sichuan specimens (One-way ANOVA, *p* < 0.01) (Table 2). Compared with the Sichuan and Guizhou specimens, the “V” shaped notch in the posterior of pre-anal pseudosegment and the knobs of anal pseudosegment were not noticeable in Tibetan specimens, and some Tibetan specimens had eye spots that could easily be overlooked (Fig. 5A).

## ﻿Discussion

### ﻿Challenges and suggestions for species diversity studies of bdelloid rotifers in China

Southern China has two biodiversity hotspots, covering tropical, subtropical, and plateau climates, with an area approximately one-third the size of Europe. However, only 36.4% of the number of Europe’s bdelloid rotifers and approximately 25% of the world’s bdelloid rotifers have been reported from China. Possibly, bdelloid rotifer species richness may be further increased in China if the following aspects are considered: First, imbalanced sampling is a problem when evaluating rotifer diversity in China. For example, poor sampling efforts in some areas, especially in Northern China such as Shanxi, Hebei, and Jilin provinces, have resulted in an underestimation of bdelloid rotifer species richness. Therefore, the diversity of bdelloid rotifers in these provinces remains unknown. The inconsistent sampling effort mainly caused the differences in species richness of bdelloid rotifers in different areas in this study. Although the sampling sites in this study included a variety of limno–terrestrial and aquatic habitats, the sampling densities in these habitats were uneven. In limno–terrestrial habitats rich in bdelloid species, the number of leaf litter and moss samples was much higher than that of lichens and soil samples, resulting in a large difference in species richness. Previous studies have identified 22, 24, and 17 bdelloid rotifers in Czech, Hungarian, and Antarctic soils, respectively (Devetter 2009; Schoell and Devetter 2013; Smykla et al. 2018). However, some species, such as *Mniobiagranulosa* Bartoš, 1940 and *M.incrassata* (Murray, 1905), which were highly abundant and common in soil habitats, were not found in this study (Devetter 2009). Fontaneto and Ricci (2006) found differences in the species composition of mosses and lichens, and the estimates of lichen species richness were much higher than those of moss habitats, indicating that lichen habitats contain a large number of rare species. Similar results were obtained in Turkey and the middle Arctic Spitsbergen (Kaya et al. 2010; Kaya and Erdoğan 2015).

Rare species are often found in both unique and overlooked habitats. Studies on the diversity of bdelloid rotifers in less-investigated environments should be conducted to accurately assess the distribution of rare species. In this study, *M.pinnigera* was found in moss in dolines and has been recorded in four countries (Uganda, South Africa, Indonesia, and Korea) in Asia and Africa (Song and Lee 2020). *Bradyscelahoonsooi* is a rare species discovered by Song (2015) in Korea, and has not been reported since then. Moreover, *E.hamata* from the island stream habitat was thought to be a parasite of *Gammarus* (Murray 1906) and was also found in the bed sediments of a mountain stream (Schmid-Araya 1993). This species is very rare and has mainly been found in European countries such as Scotland, Austria, France, and Italy, which belong to the Palearctic region (Donner 1965; Ferrari et al. 2023; Jersabek and Leitner 2024). This study confirmed that these rare species are distributed in the Oriental region.

Another possible reason for the underestimation of species richness in China is the lack of quantitative studies on spatio–temporal variation. Notably, the abundance of some monogonont rotifers varies seasonally, and even disappears during certain periods, which can greatly affect the assessment of local species richness (Liang et al. 2020; Yuan et al. 2020). Similarly, Devetter (2009) found that the bdelloid rotifer community structure changes significantly during the year, and species richness increases from September to May. For example, *C.cornigera* was present from July to December but disappeared in February and May. In this study, *C.cornigera* was identified in China for the first time and was never found in any other province except Tibet. Although *C.cornigera* has been reported in several biogeographic regions, it has not yet been recorded in the Oriental region. Moreover, specific relationships with environmental factors, such as climate (Qian et al. 2021), altitude (Devetter et al. 2017), latitude, and longitude (Fontaneto et al. 2015), should also be considered to increase the diversity of bdelloid rotifers, especially in Oriental, a tropical and subtropical region with a wide latitude and longitude span.

In addition to morphological identification, molecular methods can reveal a great deal of hidden diversity in bdelloid rotifers and can recognise many morphologically indistinguishable subspecies or genetic clusters adapted to a particular region or environment (Fontaneto et al. 2019). This technique has been developed and applied to study bdelloid rotifer diversity in many areas, including Antarctica (Iakovenko et al. 2015; Cakil et al. 2021). However, in China, relevant studies are limited to the monogonont species (Deng et al. 2022), and the hidden diversity of bdelloid rotifers in China remains unexplored. Therefore, a phylogenetic study of bdelloid rotifers in some special areas and habitats, such as the Tibetan Plateau, will provide important basic data for the study of biogeography on a global scale and fill the knowledge gap in China.

### ﻿Biogeographic distribution of bdelloid rotifers in different areas in China

As studies on the species diversity of microscopic organisms expand in both temporal and spatial scales, acquiring more species occurrence data aids researchers in gaining a clearer understanding of the biogeographic distribution of species, which indicates the presence of endemism in microscopic organisms, such as rotifers (Fontaneto et al. 2008; Iakovenko et al. 2015). We found four endemic species (collected from only one biogeographical area) on a global scale, accounting for 2.84% of all bdelloid rotifers in China (Segers 2007). These endemic species include three Oriental species and one Palearctic species. These results complement those of the bdelloid rotifer fauna from the less-studied Oriental region (Segers 2007; Zeng et al. 2020). In Thailand, Jaturapruek et al. (2018) recorded one tropical Oriental species and two species with narrow geographical distributions. This result is consistent with the general consensus among rotifer scientists that most bdelloid rotifers are widespread, although there are some endemic species (Fontaneto et al. 2008; Iakovenko et al. 2015). Restricted biogeographic distributions may be due to different reasons, such as insufficient sampling (Fontaneto et al. 2012, 2015) or environmental filtering effects (Le Bagousse‐Pinguet et al. 2017).

The Tibetan Plateau is considered the third pole of the Earth. Its harsh environmental conditions, such as high altitude, low temperature, low oxygen concentration, and strong ultraviolet rays, strongly affect the living organisms and promote the formation of many unique species (Deng et al. 2022). In this study, we found some rare species occurring only in Tibet, such as *Adinetabarbata* Janson, 1893 and *Habrotrochaelusa* Milne, 1916. Among these species, *H.quinquedens* De Koning, 1947 exists only in the Palearctic (Segers 2007). In addition, morphological analysis revealed the intraspecific differentiation of *P.chinensis* sp. nov. in different regions and the Tibetan species is the smallest. Deng et al. (2022) identified a new mitochondrial clade of the monogonont rotifer *Brachionusplicatilis* Müller, 1786 in Tibet based on DNA taxonomy and morphological evidence. This clade is restricted to the Qinghai–Tibet Plateau, and the body size of the species in this clade is larger than that of other clades in Inner Mongolia and Northeast China. Differences in body size are attributed to phenotypic plasticity in response to environmental stress (Deng et al. 2022). Therefore, we often find unique species adapted to local habitats in extreme environments and regions (Iakovenko et al. 2015).

Currently, biogeographical research on bdelloid rotifers in China is limited to the morphological level (Zeng et al. 2020). Notably, morphological methods may not have a high resolution for cryptic species and tend to overlook entities that are morphologically similar but genetically divergent (Kaya et al. 2009). For example, five bdelloid species found in Antarctica have morphological characteristics that are indistinguishable from already known cosmopolitan species; however, they have been identified as new species endemic to Antarctica using DNA taxonomy (Iakovenko et al. 2015). Through a comparative analysis of phylogeography, Cakil et al. (2021) found that the genetic diversity of Antarctic bdelloids is significantly shallower than that of other parts of the world. Fontaneto et al. (2008) collected COI gene sequences of *Adineta* and *Rotaria* species worldwide and revealed the biogeographic distribution pattern of bdelloid rotifers through molecular phylogenetic methods. Although most species were widely distributed, endemic species were also reported. In addition, integrated morphological and molecular analyses can better reveal the genetic differentiation within rotifers in different regions on a fine scale (Deng et al. 2022; Yang et al. 2022). Therefore, to fully understand the diversity and biogeographic distribution of Chinese rotifers and to discover the endemic species in China, integrated morphological and molecular methods should be used in the future.

In general, the more adaptable a species, the wider its distribution. However, some species, such as *A.beysunae*, show exception to this rule. In this study, *A.beysunae* was found only in the leaf litter habitat but was distributed at nine sites, which is consistent with previous results (Örstan 2018; Zeng et al. 2020; Wang et al. 2023). This strong habitat preference suggests that certain environmental factors are necessary for the survival of bdelloid rotifers. Rapidly developing molecular techniques, such as transcriptomics and metabolomics, can help us explore the response of *A.beysunae* to specific environmental factors in leaf litter habitats at the genetic level (Gribble and Welch 2017). Future studies on the relationship between bdelloid rotifers and biological and abiotic factors in the environment may solve the problem of habitat selectivity and adaptability of bdelloid rotifers, providing new insights into the factors driving the biogeographical distribution patterns of bdelloid rotifers and their relationships with ecological niches.

## ﻿Conclusions

In this study, we summarised the diversity and distribution of bdelloid rotifers in different areas and habitats of China, providing important data for the theory of the biogeography of global microscopic organisms. The results indicate a high species diversity of bdelloid rotifers in China. Furthermore, two new species were described. Three newly recorded genera and 26 newly recorded species were found, with four species with restricted distribution. This study proposed suggestions for diversity studies of bdelloid rotifers and recommended that more research should be focused on molecular analysis of bdelloid rotifer diversity and biogeography.

## Supplementary Material

XML Treatment for Adineta jinan

XML Treatment for Philodina chinensis

## ﻿Additional information

### Data availability

The data are available from the corresponding author on reasonable request.

## Supplementary materials

Supplementary material 1 Supplementary information Data type docx Explanation note **table S1.** Information of sampling locality in this study. **table S2.** Distribution of bdelloid rotifers identified in this study. https://binary.pensoft.net/file/1260144This dataset is made available under the Open Database License (http://opendatacommons.org/licenses/odbl/1.0/). The Open Database License (ODbL) is a license agreement intended to allow users to freely share, modify, and use this Dataset while maintaining this same freedom for others, provided that the original source and author(s) are credited.Wenbo Wang, Yufeng Yang, Aydin Örstan, Zhili He, Qing Wang
