# Supplementary material for: ﻿Species diversity of bdelloid rotifer (Rotifera, Bdelloidea) in different areas in China, with a description of two new species
Source: Zookeys. 2025 Feb 24;1229:1–23. doi: 10.3897/zookeys.1229.113385 (PMC11876980; doi:10.3897/zookeys.1229.113385)
Supplement: Supplementary material 1 — Supplementary information [file zookeys-1229-001_article-113385__-s001.docx]

| **Table S1 Information of sampling locality in this study** | | | | |
| --- | --- | --- | --- | --- |
| **Locality codes** | **Sites** | **Habitat** | **Latitude** | **Longitude** |
| CD8 | Changde | moss | 29.15190511°N | 111.7188263°E |
| CD7 | Changde | bamboo leaf litter | 29.15957585°N | 111.70918°E |
| CD19 | Changde | leaf litter | 28.95440892°N | 111.6951948°E |
| CD22 | Changde | moss | 28.95640326°N | 111.6948164°E |
| CD26 | Changde | moss | 29.01354265°N | 111.6969657°E |
| CD21 | Changde | bamboo leaf litter | 28.95629156°N | 111.6946468°E |
| CD4 | Changde | bamboo leaf litter | 29.16811072°N | 111.7113189°E |
| CD36 | Changde | bamboo leaf litter | 29.04865118°N | 111.673212°E |
| CD33 | Changde | moss | 29.0509609°N | 111.6800731°E |
| CD15 | Changde | leaf litter | 29.04979683°N | 111.5948762°E |
| CD20 | Changde | moss | 28.95462481°N | 111.695106°E |
| CD16 | Changde | moss | 29.04920437°N | 111.595354°E |
| CD17 | Changde | moss | 29.04839919°N | 111.5961099°E |
| CD24 | Changde | leaf litter | 28.98890042°N | 111.6848112°E |
| CD2 | Changde | moss | 29.17275024°N | 111.7095384°E |
| CD29 | Changde | bamboo leaf litter | 29.03612817°N | 111.7034044°E |
| CD10 | Changde | moss | 29.05416161°N | 111.5937636°E |
| CD37 | Changde | bamboo leaf litter | 29.04476767°N | 111.6721857°E |
| CD34 | Changde | moss | 29.05116511°N | 111.6715216°E |
| CD3 | Changde | bamboo leaf litter | 29.17228016°N | 111.7100286°E |
| 19-CD9 | Changde | moss | 29.01326676°N | 111.69701191°E |
| 18-C1 | Changde | water | 29.05055556°N | 111.70694444°E |
| 18-C2 | Changde | water | 29.05388889°N | 111.69194444°E |
| 18-C3 | Changde | bamboo leaf litter | 29.05013319°N | 111.67205243°E |
| 19-CD40 | Changde | leaf litter | 29.04891774°N | 111.68928408°E |
| 19-CD23 | Changde | leaf litter | 29.05117439°N | 111.67152029°E |
| 19-CD27 | Changde | moss | 29.02965444°N | 111.67091447°E |
| 19-CD33 | Changde | aquatic plant | 29.10682006°N | 111.72087017°E |
| 19-CD39 | Changde | aquatic plant | 29.04890723°N | 111.68877938°E |
| 19-CD38 | Changde | bamboo leaf litter | 29.04953176°N | 111.68695946°E |
| 19-CD1 | Changde | leaf litter | 28.95442223°N | 111.69521791°E |
| 19-CD2 | Changde | moss on tree | 28.95461095°N | 111.69508029°E |
| 19-CD3 | Changde | bamboo leaf litter | 28.95623847°N | 111.69468281°E |
| 19-CD4 | Changde | leaf litter | 28.95606271°N | 111.69460633°E |
| 19-CD5 | Changde | leaf litter | 28.95624077°N | 111.69387228°E |
| 19-CD6 | Changde | leaf litter | 28.99032947°N | 111.68287882°E |
| 19-CD7 | Changde | moss | 28.99058641°N | 111.68248884°E |
| 19-CD8 | Changde | leaf litter | 28.98880254°N | 111.68495105°E |
| 19-CD10 | Changde | moss | 29.01478831°N | 111.69918353°E |
| 19-CD11 | Changde | moss | 29.01683582°N | 111.69671399°E |
| 19-CD12 | Changde | moss | 29.0193635°N | 111.70319793°E |
| 19-CD13 | Changde | moss on tree | 29.0191463°N | 111.70343492°E |
| 19-CD14 | Changde | leaf litter | 29.03672251°N | 111.70299291°E |
| 19-CD15 | Changde | moss on rock | 29.04494224°N | 111.59707931°E |
| 19-CD16 | Changde | moss on tree | 29.04848002°N | 111.59591272°E |
| 18-C4 | Changde | water | 29.02305556°N | 111.69722222°E |
| 18-C5 | Changde | water | 29.1175°N | 111.71361111°E |
| 18-C6 | Changde | water | 28.9675°N | 111.71416667°E |
| 18-C7 | Changde | water | 29.02333333°N | 111.69666667°E |
| CS1 | Changsha | moss on tree | 28.20992999°N | 112.99987000°E |
| CS2 | Changsha | moss on tree | 28.19684700°N | 112.94978600°E |
| CS3 | Changsha | bamboo leaf litter | 28.18211052°N | 112.93550348°E |
| 8-BY1-S | Guangzhou | leaf litter | 23.16933593°N | 113.29905517°E |
| 8-BY2-S | Guangzhou | leaf litter | 23.16933593°N | 113.29905517°E |
| 8-BY3-S | Guangzhou | leaf litter | 23.16933593°N | 113.29905517°E |
| 8-BY1-B | Guangzhou | leaf litter | 23.16933593°N | 113.29905517°E |
| 8-BY2-B | Guangzhou | leaf litter | 23.16933593°N | 113.29905517°E |
| 8-BY3-B | Guangzhou | leaf litter | 23.16933593°N | 113.29905517°E |
| 9-BY1-S | Guangzhou | leaf litter | 23.16933593°N | 113.29905517°E |
| 9-BY2-S | Guangzhou | leaf litter | 23.16933593°N | 113.29905517°E |
| 9-BY3-S | Guangzhou | leaf litter | 23.16933593°N | 113.29905517°E |
| 9-BY1-B | Guangzhou | leaf litter | 23.16933593°N | 113.29905517°E |
| 9-BY2-B | Guangzhou | leaf litter | 23.16933593°N | 113.29905517°E |
| 9-BY3-B | Guangzhou | leaf litter | 23.16933593°N | 113.29905517°E |
| 10-BY1-S | Guangzhou | leaf litter | 23.16933593°N | 113.29905517°E |
| 10-BY2-S | Guangzhou | leaf litter | 23.16933593°N | 113.29905517°E |
| 10-BY3-S | Guangzhou | leaf litter | 23.16933593°N | 113.29905517°E |
| 10-BY1-B | Guangzhou | leaf litter | 23.16933593°N | 113.29905517°E |
| 10-BY2-B | Guangzhou | leaf litter | 23.16933593°N | 113.29905517°E |
| 10-BY3-B | Guangzhou | leaf litter | 23.16933593°N | 113.29905517°E |
| HZ1 | Guangzhou | aquatic plant | 23.07362302°N | 113.32035691°E |
| HZ2 | Guangzhou | aquatic plant | 23.0734498°N | 113.32053186°E |
| HZ3 | Guangzhou | aquatic plant | 23.07276948°N | 113.31947761°E |
| HZ4 | Guangzhou | leaf litter | 23.07081626°N | 113.31994764°E |
| HZ5 | Guangzhou | leaf litter | 23.07028319°N | 113.32096325°E |
| HZ6 | Guangzhou | bamboo leaf litter | 23.06719153°N | 113.3310315°E |
| JNU1 | Guangzhou | water inlet of pool | 23.1278686°N | 113.34781569°E |
| G1-1 | Guangzhou | leaf litter | 23.16516667°N | 113.29126667°E |
| G1-2 | Guangzhou | moss on tree | 23.16886667°N | 113.29139167°E |
| G1-3 | Guangzhou | moss on rock | 23.17113889°N | 113.29525°E |
| G1-4 | Guangzhou | lichen | 23.17069722°N | 113.29835556°E |
| G1-5 | Guangzhou | moss on rock | 23.17085278°N | 113.29838056°E |
| G1-6 | Guangzhou | moss on rock | 23.17112222°N | 113.29751389°E |
| G1-7 | Guangzhou | leaf litter | 23.17160833°N | 113.29757778°E |
| G1-8 | Guangzhou | lichen | 23.17336111°N | 113.29677778°E |
| G1-9 | Guangzhou | leaf litter | 23.17506944°N | 113.29510278°E |
| G1-10 | Guangzhou | flower | 23.17514444°N | 113.29424722°E |
| G1-11 | Guangzhou | leaf litter | 23.17635278°N | 113.29356667°E |
| G2-1 | Guangzhou | moss on rock | 23.30584722°N | 113.45350833°E |
| G2-2 | Guangzhou | moss on rock | 23.30572778°N | 113.45411389°E |
| G2-3 | Guangzhou | leaf litter | 23.30571944°N | 113.45413889°E |
| G2-4 | Guangzhou | lichen on rock | 23.30566111°N | 113.45464722°E |
| G2-5 | Guangzhou | moss on soil | 23.30643889°N | 113.45592778°E |
| G2-6 | Guangzhou | leaf litter | 23.30644722°N | 113.45598056°E |
| G2-7 | Guangzhou | moss on rock | 23.30686111°N | 113.4568°E |
| G2-8 | Guangzhou | leaf litter | 23.30705°N | 113.45931389°E |
| G2-9 | Guangzhou | moss on rock | 23.30547222°N | 113.46166111°E |
| G2-10 | Guangzhou | moss on tree | 23.30523611°N | 113.46155°E |
| G2-11 | Guangzhou | moss on soil | 23.30529722°N | 113.46150833°E |
| G2-12 | Guangzhou | leaf litter | 23.30276944°N | 113.46411111°E |
| G2-13 | Guangzhou | aquatic plant | 23.30078889°N | 113.46273611°E |
| G2-14 | Guangzhou | lichen on tree | 23.302275°N | 113.46280833°E |
| G2-15 | Guangzhou | moss on rock | 23.30157222°N | 113.46661944°E |
| G2-16 | Guangzhou | leaf litter | 23.30364444°N | 113.460775°E |
| G3-1 | Guangzhou | moss on rock | 23.17552222°N | 113.37943333°E |
| G3-2 | Guangzhou | moss on rock | 23.1761°N | 113.37916111°E |
| G3-3 | Guangzhou | leaf litter | 23.17593889°N | 113.37903333°E |
| G3-4 | Guangzhou | moss on rock | 23.17765278°N | 113.37848333°E |
| G3-5 | Guangzhou | moss on rock | 23.18451111°N | 113.37943333°E |
| G3-6 | Guangzhou | leaf litter | 23.184475°N | 113.37932222°E |
| G3-7 | Guangzhou | moss on rock | 23.18460278°N | 113.377575°E |
| G3-8 | Guangzhou | leaf litter | 23.18484167°N | 113.38202222°E |
| G3-9 | Guangzhou | lichen | 23.18456389°N | 113.38157222°E |
| G3-10 | Guangzhou | moss | 23.18455833°N | 113.38151389°E |
| G3-11 | Guangzhou | leaf litter | 23.18460278°N | 113.37943333°E |
| G3-12 | Guangzhou | flower | 23.18405278°N | 113.381325°E |
| G3-13 | Guangzhou | aquatic plant | 23.18401667°N | 113.38120278°E |
| G3-14 | Guangzhou | Stream | 23.18415556°N | 113.38146667°E |
| G3-15 | Guangzhou | moss on rock | 23.18360833°N | 113.38088611°E |
| G3-16 | Guangzhou | leaf litter | 23.18367778°N | 113.38099444°E |
| G3-17 | Guangzhou | moss on soil | 23.18014167°N | 113.37926944°E |
| G3-18 | Guangzhou | leaf litter | 23.19674167°N | 113.37934722°E |
| L1-1 | Shenzhen | leaf litter | 22.57974496°N | 114.1793°E |
| L1-2 | Shenzhen | leaf litter | 22.5793178°N | 114.17966359°E |
| L1-3 | Shenzhen | leaf litter | 22.57907661°N | 114.18070395°E |
| L1-4 | Shenzhen | leaf litter | 22.58032973°N | 114.17843297°E |
| L1-5 | Shenzhen | leaf litter | 22.57884231°N | 114.18118214°E |
| L2-1 | Shenzhen | leaf litter | 22.57894409°N | 114.17923825°E |
| L2-2 | Shenzhen | leaf litter | 22.57863747°N | 114.17978175°E |
| L2-3 | Shenzhen | leaf litter | 22.57861657°N | 114.17984674°E |
| L2-4 | Shenzhen | leaf litter | 22.57853056°N | 114.1801454°E |
| L2-5 | Shenzhen | leaf litter | 22.57832488°N | 114.18037748°E |
| L3-1 | Shenzhen | leaf litter | 22.57783045°N | 114.17773801°E |
| L3-2 | Shenzhen | leaf litter | 22.57804863°N | 114.17787188°E |
| L3-3 | Shenzhen | leaf litter | 22.57850468°N | 114.17785283°E |
| L3-4 | Shenzhen | leaf litter | 22.57898406°N | 114.17755002°E |
| L3-5 | Shenzhen | leaf litter | 22.57929596°N | 114.17729382°E |
| M1-1 | Shenzhen | moss | 22.57980615°N | 114.17889909°E |
| M1-2 | Shenzhen | moss | 22.5792317°N | 114.17985692°E |
| M1-3 | Shenzhen | moss | 22.57871434°N | 114.17848511°E |
| M1-4 | Shenzhen | moss | 22.57877987°N | 114.18088712°E |
| M1-5 | Shenzhen | moss | 22.58019844°N | 114.178642°E |
| M2-1 | Shenzhen | moss | 22.57885611°N | 114.17920796°E |
| M2-2 | Shenzhen | moss | 22.57873599°N | 114.17975071°E |
| M2-3 | Shenzhen | moss | 22.57860237°N | 114.17994331°E |
| M2-4 | Shenzhen | moss | 22.57853056°N | 114.1801454°E |
| M2-5 | Shenzhen | moss | 22.57837365°N | 114.18047404°E |
| M3-1 | Shenzhen | moss | 22.57783045°N | 114.17773801°E |
| M3-2 | Shenzhen | moss | 22.57804863°N | 114.17787188°E |
| M3-3 | Shenzhen | moss | 22.57850468°N | 114.17785283°E |
| M3-4 | Shenzhen | moss | 22.57892821°N | 114.17763161°E |
| M3-5 | Shenzhen | moss | 22.57934495°N | 114.17721172°E |
| S1-1 | Shenzhen | soil | 22.5797149°N | 114.17910266°E |
| S1-2 | Shenzhen | soil | 22.57921167°N | 114.1799169°E |
| S1-3 | Shenzhen | soil | 22.57876153°N | 114.18077548°E |
| S1-4 | Shenzhen | soil | 22.5792516°N | 114.18039979°E |
| S1-5 | Shenzhen | soil | 22.58027958°N | 114.17802016°E |
| S2-1 | Shenzhen | soil | 22.57880838°N | 114.17928934°E |
| S2-2 | Shenzhen | soil | 22.5786917°N | 114.17974022°E |
| S2-3 | Shenzhen | soil | 22.57860237°N | 114.17994331°E |
| S2-4 | Shenzhen | soil | 22.57858808°N | 114.1800863°E |
| S2-5 | Shenzhen | soil | 22.57829354°N | 114.18054702°E |
| S3-1 | Shenzhen | soil | 22.57783045°N | 114.17773801°E |
| S3-2 | Shenzhen | soil | 22.57804863°N | 114.17787188°E |
| S3-3 | Shenzhen | soil | 22.57843811°N | 114.1778483°E |
| S3-4 | Shenzhen | soil | 22.57892821°N | 114.17763161°E |
| S3-5 | Shenzhen | soil | 22.57934495°N | 114.17721172°E |
| W1-1 | Shenzhen | water | 22.57920851°N | 114.1799837°E |
| W1-2 | Shenzhen | water | 22.58130138°N | 114.18091668°E |
| W1-3 | Shenzhen | water | 22.58121957°N | 114.18087542°E |
| W1-4 | Shenzhen | water | 22.5811829°N | 114.18077553°E |
| W1-5 | Shenzhen | water | 22.5811829°N | 114.18077553°E |
| W2-1 | Shenzhen | water | 22.58221244°N | 114.17231639°E |
| W2-2 | Shenzhen | water | 22.58221244°N | 114.17231639°E |
| W2-3 | Shenzhen | water | 22.58221244°N | 114.17231639°E |
| W2-4 | Shenzhen | water | 22.58278552°N | 114.17336248°E |
| W2-5 | Shenzhen | water | 22.58278552°N | 114.17336248°E |
| W3-1 | Shenzhen | water | 22.57899836°N | 114.17756756°E |
| W3-2 | Shenzhen | water | 22.57907572°N | 114.17750893°E |
| W3-3 | Shenzhen | water | 22.57899836°N | 114.17756756°E |
| W3-4 | Shenzhen | water | 22.57921526°N | 114.17737788°E |
| W3-5 | Shenzhen | water | 22.5792998°N | 114.17733864°E |
| XH1 | Shenzhen | moss | 22.57579982°N | 114.1772347°E |
| XH7 | Shenzhen | moss | 22.57474849°N | 114.17700332°E |
| XH16 | Shenzhen | moss | 22.57474849°N | 114.17700332°E |
| XH19 | Shenzhen | moss | 22.57474849°N | 114.17700332°E |
| XH27 | Shenzhen | moss | 22.57474849°N | 114.17700332°E |
| SZ2 | Shenzhen | banyan leaf litter | 22.56654722°N | 114.07368055°E |
| XH35 | Shenzhen | moss | 22.57474849°N | 114.17700332°E |
| FS1 | Foshan | leaf litter | 22.81557222°N | 113.14416944°E |
| FS2 | Foshan | bamboo leaf litter | 22.81394167°N | 113.14888333°E |
| YX1 | Yongxing island | leaf litter | 16.83248300°N | 112.35016199°E |
| YX2 | Yongxing island | moss | 16.83478999°N | 112.34371899°E |
| YX3 | Yongxing island | bamboo leaf litter | 16.83478999°N | 112.34371899°E |
| WS3 | Wanshan island | moss | 21.93539331°N | 113.72618371°E |
| WS6 | Wanshan island | leaf litter | 21.93600871°N | 113.73221893°E |
| WS7 | Wanshan island | moss | 21.93977106°N | 113.73339807°E |
| WS19 | Wanshan island | moss | 21.93125276°N | 113.72510823°E |
| WLD1 | Wailingding island | moss on tree | 22.09962405°N | 114.03081303°E |
| WLD6 | Wailingding island | moss | 22.10059059°N | 114.04220317°E |
| WLD7 | Wailingding island | bamboo leaf litter | 22.10059844°N | 114.04235972°E |
| QA3 | Qiao island | leaf litter | 22.43366664°N | 113.63898062°E |
| QA4 | Qiao island | moss | 22.43366664°N | 113.63898062°E |
| QA8 | Qiao island | moss | 22.4305594°N | 113.63949235°E |
| QA1 | Qiao island | leaf litter | 22.42553823°N | 113.6312006°E |
| QA2 | Qiao island | sediment | 22.42706278°N | 113.62985355°E |
| QA5 | Qiao island | sediment | 22.42706278°N | 113.62985355°E |
| QA6 | Qiao island | sediment | 22.42706278°N | 113.62985355°E |
| QA7 | Qiao island | sediment | 22.42706278°N | 113.62985355°E |
| QA9 | Qiao island | sediment | 22.42706278°N | 113.62985355°E |
| NA1 | Nanao island | moss near stream | 23.44174852°N | 117.09469237°E |
| NA5 | Nanao island | aquatic plant in Stream | 23.43409463°N | 117.08788008°E |
| NA6 | Nanao island | stream | 23.44174852°N | 117.09469237°E |
| NA7 | Nanao island | moss on rock | 23.41801111°N | 117.02998889°E |
| NA8 | Nanao island | moss on soil | 23.47533889°N | 117.06790278°E |
| NA9 | Nanao island | leaf litter | 23.47507778°N | 117.06821111°E |
| NA10 | Nanao island | moss on soil | 23.47658889°N | 117.10521111°E |
| NA11 | Nanao island | moss on soil | 23.47933333°N | 117.1156°E |
| NA12 | Nanao island | leaf litter | 23.45503611°N | 117.125375°E |
| MW3 | Miaowan island | leaf litter | 21.86786424°N | 114.02107776°E |
| MW6 | Miaowan island | leaf litter | 21.85930881°N | 114.01065606°E |
| MW10 | Miaowan island | leaf litter | 21.82445188°N | 113.96782627°E |
| GS3 | Guishan island | leaf litter | 22.13499006°N | 113.83601164°E |
| ZH1 | Zhuhai | bamboo leaf litter | 22.26447778°N | 113.577525°E |
| ZH2 | Zhuhai | leaf litter | 22.29689342°N | 113.57732427°E |
| ZH3 | Zhuhai | leaf litter | 22.29689342°N | 113.57732427°E |
| ZH4 | Zhuhai | leaf litter | 22.29689342°N | 113.57732427°E |
| ZH5 | Zhuhai | leaf litter | 22.27621290°N | 113.58812990°E |
| ZH6 | Zhuhai | leaf litter | 22.27621290°N | 113.58812990°E |
| ZH7 | Zhuhai | leaf litter | 22.26099965°N | 113.57085936°E |
| ZH8 | Zhuhai | leaf litter | 22.26099965°N | 113.57085936°E |
| ZH9 | Zhuhai | leaf litter | 22.25612765°N | 113.58572864°E |
| HK1 | Haikou | leaf litter | 20.04517057°N | 110.21085467°E |
| HK2 | Haikou | bamboo leaf litter | 19.90476446°N | 110.31323084°E |
| HK3 | Haikou | bamboo leaf litter | 20.05674800°N | 110.32873799°E |
| HK4 | Haikou | moss on rock | 19.97648699°N | 110.22620299°E |
| GL1 | Guilin | bamboo leaf litter | 25.2666844°N | 110.29581708°E |
| GL2 | Guilin | moss on rock | 25.2678647°N | 110.29511685°E |
| GL3 | Guilin | moss | 25.23885318°N | 110.30382598°E |
| GL4 | Guilin | bamboo leaf litter | 25.05264622°N | 110.31729529°E |
| GL5 | Guilin | moss | 25.05542925°N | 110.31635003°E |
| GL6 | Guilin | bamboo leaf litter | 24.80854259°N | 110.39904676°E |
| GL7 | Guilin | bamboo leaf litter | 25.25296858°N | 110.27483455°E |
| GL8 | Guilin | moss | 24.99066899°N | 110.13840200°E |
| GL9 | Guilin | soil | 24.99066899°N | 110.13840200°E |
| GL10 | Guilin | moss | 24.99066899°N | 110.13840200°E |
| GL11 | Guilin | moss | 24.99066899°N | 110.13840200°E |
| GL12 | Guilin | moss | 24.99066899°N | 110.13840200°E |
| GL13 | Guilin | moss | 24.99066899°N | 110.13840200°E |
| GL14 | Guilin | leaf litter | 24.77645563°N | 110.49098399°E |
| GL15 | Guilin | moss | 24.77645563°N | 110.49098399°E |
| GL16 | Guilin | moss on soil | 25.2524983°N | 110.2733124°E |
| GL17 | Guilin | moss on rock | 25.08831702°N | 110.25458584°E |
| NN1 | Nanning | bamboo leaf litter | 22.82788114°N | 108.3312795°E |
| NN2 | Nanning | moss on rock | 22.78129334°N | 108.38780671°E |
| WZ1 | Weizhou island | bamboo leaf litter | 21.05050723°N | 109.13363374°E |
| WZ2 | Weizhou island | moss on rock | 21.05091279°N | 109.13665781°E |
| WZ3 | Weizhou island | moss on rock | 21.05091279°N | 109.13665781°E |
| LZ5 | Linzhi | bamboo leaf litter | 29.64172037°N | 94.36012648°E |
| LZ10 | Linzhi | moss | 29.69966555°N | 94.34115008°E |
| LZ13 | Linzhi | moss on rock | 29.71842212°N | 94.31537766°E |
| LZ14 | Linzhi | moss | 29.69951569°N | 94.34130958°E |
| LZ16 | Linzhi | leaf litter | 29.70051656°N | 94.34118946°E |
| LZ17 | Linzhi | moss | 29.70089253°N | 94.34100132°E |
| 318 1# | Linzhi | moss | 29.56061912°N | 94.52248044°E |
| 318 3# | Linzhi | moss on tree | 29.56513957°N | 94.56045663°E |
| 318 4# | Linzhi | leaf litter | 29.56512724°N | 94.56011403°E |
| 318 5# | Linzhi | moss on tree | 29.55844656°N | 94.55398424°E |
| 318 12# | Linzhi | lichen | 30.03435629°N | 95.27066653°E |
| KD1 | Linzhi | bamboo leaf litter | 29.75603779°N | 94.17015244°E |
| KD2 | Linzhi | bamboo leaf litter | 29.75714712°N | 94.17004653°E |
| KD4 | Linzhi | moss on tree | 29.75718044°N | 94.17003397°E |
| KD6 | Linzhi | moss on rock | 29.76144596°N | 94.17075403°E |
| LZ1 | Linzhi | moss | 29.63114686°N | 94.36292937°E |
| LZ2 | Linzhi | moss | 29.63114185°N | 94.36183989°E |
| LZ4 | Linzhi | moss | 29.64055174°N | 94.36010231°E |
| BDL2 | Lasa | moss | 29.65835693°N | 91.12450386°E |
| LS3 | Lasa | leaf litter | 29.66424838°N | 91.10024332°E |
| LS4 | Lasa | leaf litter | 29.66429473°N | 91.09634799°E |
| LS5 | Lasa | bamboo leaf litter | 29.65772449°N | 91.11692607°E |
| LS7 | Lasa | moss | 29.65714144°N | 91.11927254°E |
| LS9 | Lasa | moss | 29.65773979°N | 91.12134733°E |
| DX1 | Dangxiong | moss on soil | 30.46674212°N | 91.08491279°E |
| DX4 | Dangxiong | moss on rock | 30.77342088°N | 90.86214227°E |
| DX5 | Dangxiong | plant | 30.67911475°N | 91.09926954°E |
| BM2 | Bomi | moss on rock | 29.87819525°N | 95.73404477°E |
| BM5 | Bomi | moss on rock | 29.87944986°N | 95.72584603°E |
| BM6 | Bomi | leaf litter | 29.89003706°N | 95.53273225°E |
| BM8 | Bomi | moss on tree | 29.89009711°N | 95.53278483°E |
| BM9 | Bomi | moss | 29.90498074°N | 95.47963379°E |
| BM10 | Bomi | leaf litter | 29.90483581°N | 95.47970461°E |
| GY1 | Guiyang | bamboo leaf litter | 26.32935711°N | 106.68818011°E |
| GY2 | Guiyang | leaf litter | 26.33154992°N | 106.68935322°E |
| GY3 | Guiyang | leaf litter | 26.33377669°N | 106.68854155°E |
| ST3 | Anshun | leaf litter | 26.018006°N | 105.681564°E |
| ST4 | Anshun | bamboo leaf litter | 26.012703°N | 105.6856°E |
| ST5 | Anshun | moss on tree | 25.406111°N | 105.673922°E |
| ST6 | Anshun | moss | \ | \ |
| SCCD1 | Chengdu | leaf litter | 30.7104°N | 104.1063°E |

| **Table S2 Distribution of bdelloid rotifers identified in this study** | |
| --- | --- |
| **Species** | **Locality codes** |
| *Adineta acuticornis* Haigh, 1967 | CD8, CD7, CD19, CD22, CD4, CD33, CD20, CD16, CD17, YX2, YX3, GL2, GL6, GL7, NN1, WZ1, LZ5, L1-4, L2-2, L2-3, L2-4, L2-5, L3-2, L3-3 |
| *Adineta barbata* Janson, 1893 | 318 3# |
| *Adineta beysunae* Örstan, 2018 | 18-C3, CD7, CD19, CD21, CD4, CD36, CD15, CD24, CD29, CD37, CD3, HZ4, HZ5, HZ6, SZ2, FS1, 9-BY2-S, FS2, GY1, ST3, ST4, GL1, GL4, GL6, GL7, NN1, HK2, L1-1, L1-2, L1-3, L1-4, L1-5, L2-1, L2-2, L2-3, L2-5, L3-1, L3-2, L3-3, G1-7, G1-11, G-16, G3-3, G3-8 |
| *Adineta cuneata* Milne, 1916 | 19-CD9, CD8, CD19, CD33, CD15, CD2, CD29, 10-BY1-S, FS1, ZH1, ZH3, ZH6, ZH7, XH16, XH7, XH27, XH1, ST3, ST6, GL5, L1-2, L2-2, L2-3, L2-5, L3-1, L3-2, L3-4, M1-1, M1-5, M2-3, M2-5, M3-1, M3-3, G2-11, G3-16 |
| *Adineta jinan* sp. nov. | L2-2, L2-3, M2-1 |
| *Adineta oculata* (Milne, 1886) | SZ1 |
| *Adineta ricciae* Segers & Shiel, 2005 | 19-CD39 |
| *Adineta steineri* Bartoš, 1951 | 10-BY2-S |
| *Adineta vaga* (Davis, 1873) | 18-C1, 18-C3, CD17 CD37 19CD, FS1, XH19, YX1, WS7, GY1, ST4, GL5, GL6, NN2, WZ1, LZ5, LS5, BM5, DX4, BM10, HK1, L2-3, M1-4, M2-1 |
| *Adineta vaga minor* Bryce, 1893 | 19-CD23, CD4, 10-BY2-S, 10-BY3-B |
| *Bradyscela clauda* (Bryce, 1893) | 18-C3, CD21 CD36, 10-BY3-S, FS2, ST4, L1-1, L1-2, L3-2, L3-4 |
| *Bradyscela hoonsooi* Song & Min, 2015 | L1-4, L2-3, L2-5, M2-1, M3-1 |
| *Habrotrocha angusticollis* (Murray, 1905) | XH27, L1-1, L1-4, L2-2, M2-2 |
| *Habrotrocha bidens* (Gosse, 1851) | 19-CD39, CD8 CD7 CD19 CD33 CD15, 10-BY3-S, SZ2, FS1, ZH1, ZH2, ZH5, ZH8, ZH9, XH1, XH16, XH19, XH27, GY1, GL14, GL3, GL4, WZ1, LS5, LZ16, HK3, HK4, L2-1, L2-3, L2-4, L2-5, M2-2, M3-4, S1-4, G1-2 |
| *Habrotrocha constricta* (Dujardin, 1841) | 18-C3, CD15 CD3, 10-BY3-S, 10-BY3-B, FS1, XH16, GY1, LS5, 318 3#, L1-4, L2-5, L3-2, L3-3, L3-5, S2-1, G1-1, G1-11, G2-12 |
| *Habrotrocha diarthrantenna* De Koning, 1947 | CD19 |
| *Habrotrocha elusa* Milne, 1916* | LS5 |
| *Habrotrocha flava* Bryce, 1915 | CD7 CD19 CD26 CD21 CD4 CD36 CD33 CD17 CD3, 8-BY1-S, 9-BY1-S, 9-BY2-S, 9-BY3-S, 10-BY1-B, 10-BY2-B, ZH1, ZH7, XH19, XH27, YX3, QA1, QA3, ST4, GL6, GL17, 318 5#, 318 3#, 318 4#, L1-1, L1-2, L1-3, L1-4, L1-5, L2-1, L2-2, L2-3, L2-4, L2-5, L3-2, L3-3, L3-4, L3-5, M1-3, M1-4, M2-1, M2-2, M3-2, M3-3, M3-4, M3-5, S1-3 |
| *Habrotrocha filum* Donner, 1949* | L3-1 |
| *Habrotrocha gracilis* Montet, 1915* | M1-3, M1-4, M1-5, M2-2, M2-3, M2-4, M2-5, M3-2, M3-3 |
| *Habrotrocha humilis* Schulte, 1954 | CD8 |
| *Habrotrocha ligula* Bryce, 1913 | FS1, BM6, 318 1#, DX5, S1-3 |
| *Habrotrocha ligula ligula* Bryce, 1913 | CD19, FS1, MW10 |
| *Habrotrocha lata* (Bryce, 1892)* | S3-4 |
| *Habrotrocha nodosa* (Murray, 1906)* | MW10, ST3, ST4, L1-1, L1-5, L2-3 |
| *Habrotrocha pavida* Bryce, 1915* | 19-CD38, M1-4, M1-5, M3-2, M3-4 |
| *Habrotrocha parvipes* Donner, 1951 | CD4 |
| *Habrotrocha quinquedens doornensis* De Koning, 1947* | DX5 |
| *Habrotrocha rosa* Donner, 1949 | 19-CD23, CD36, GY1, WZ1, DX4, LS3, LS4, L1-2, L2-4, L2-5, L3-5 |
| *Habrotrocha rara* Donner, 1949 | CD2 |
| *Habrotrocha scepanotrochoides* De Koning, 1947* | 10-BY3-B, L2-3 |
| *Habrotrocha stenostephana* Schulte, 1954* | L3-2, L3-5, M1-4 |
| *Habrotrocha thienemanni* Hauer, 1924 | 18-C3 |
| *Habrotrocha thienemanni rubella* Donner, 1951* | MW3 |
| *Habrotrocha tranquilla* Milne, 1916 | CD37 |
| *Habrotrocha tripus* (Murray, 1907)* | WS6, NN2, LZ16, L1-1, L3-4, S3-1 |
| *Habrotrocha visa* Donner, 1954* | XH1, M3-4, M3-5 |
| *Otostephanos auriculatus* (Murray, 1911)* | L1-1, L1-4, L2-2 |
| *Otostephanos auriculatus bilobatus* Hauer, 1939* | 10-BY2-S |
| *Otostephanos donneri* Bartoš, 1959 | CD26, 10-BY2-B, S2-4 |
| *Otostephanos regalis* Milne, 1916 | 10-BY1-B, HZ5, HZ6, GL15, GL5, GL17, KD4, L1-1, L2-4, M1-5, G1-7 |
| *Otostephanos torquatus* (Bryce, 1913) | NN2 |
| *Otostephanos torquatus torquatus* (Bryce, 1913) | 19-CD9, 19-CD23, XH1, XH7, XH16, XH19, GL1 |
| *Otostephanos torquatus amoenus* Milne, 1916 | CD15, GL16, GL7, GL17, GL8, BM2 |
| *Scepanotrocha semitecta* Donner, 1951 | CD3 |
| *Rotaria citrina* (Ehrenberg, 1838) | W1-1, W1-2, W1-3, W1-4, W2-2, W2-3, W2-4, W2-5, W3-2, W3-3, W3-4, W3-5 |
| *Rotaria macroceros* (Gosse, 1851) | W2-4 |
| *Rotaria montana* (Murray, 1911)* | 318 5#, BM9, LS9, LZ10 |
| *Rotaria rotatoria* (Pallas, 1766) | 19-CD33, 18-C1, 18-C4, 18-C5, 18-C6, 18-C7, HZ3, W1-1, W1-2, W1-3, W1-4, W1-5, G2-13 |
| *Rotaria sordida* (Western, 1893) | CD8 CD7 CD19 CD26 CD21 CD4 CD33 CD16 CD2 CD29 CD37 CD3, CS1, CS2, CS3, ZH1, ZH8, XH16, XH7, XH27, XH1, YX1, YX3, WS19, WS7, WLD7, QA4, QA3, ST4, ST6, GL13, GL1, GL2, GL3, GL5, GL4, GL7, GL17, GL8, GL9, WZ1, LZ13, BM5, LZ14, 318 12#, LZ17, KD2, HK3, L1-1, L1-2, L1-5, L2-2, L3-3, M1-1, M1-2, M1-3, M1-4, M1-5, M2-1, M2-2, M2-3, M2-4, M2-5, M3-1, M3-2, M3-3, M3-4, M3-5, S1-1, S1-5, S2-1, S2-2, S2-3, S2-4, S3-1, S3-2, S3-3, S3-4, S3-5 |
| *Rotaria tardigrada* (Ehrenberg, 1830) | CD26, HZ1, HZ2, HZ3, W1-4, W2-1, W2-3, W2-4, W2-5 |
| *Rotaria tridens* (Montet, 1915) | W2-3 |
| *Dissotrocha aculeata* (Ehrenberg, 1830) | W2-3, W2-5 |
| *Dissotrocha macrostyla* (Ehrenberg, 1838) | 18-C7, NA6 |
| *Pleuretra brycei* (Weber, 1898) | NA1, NA7, NA8, M1-1, M1-2, M1-5, M2-3, M2-5 |
| *Embata hamata* (Murray, 1906)* | NA6 |
| *Macrotrachela aculeata* Milne, 1886 | CD4 CD36, GY1, ST4, GL8, NN1, M2-4 |
| *Macrotrachela ambigua* Donner, 1965 | 19-CD38, CD19, L2-4, L2-5, L3-4 |
| *Macrotrachela brevilabris* De Koning, 1947* | GL1, BM5, DX4, M3-3, M3-4, M3-5 |
| *Macrotrachela concinna* (Bryce, 1912)* | QA3, GL7 |
| *Macrotrachela decora* (Bryce, 1912)* | M1-5, S1-1, S1-4, S1-5, S2-2, S2-4, S2-5, S3-3, S3-4, S3-5 |
| *Macrotrachela ehrenbergii* (Janson, 1893) | 19-CD23, 19-CD38, CD4 CD36 CD2 CD3, CS1, 10-BY1-B, 10-BY3-B, KD4, BM9, BM10, L1-3, L2-2, L2-3, L3-5 |
| *Macrotrachela formosa* (Murray, 1906)* | WLD7, KD6 |
| *Macrotrachela habita* (Bryce, 1894) | CD16 CD17 CD10 CD3, 10-BY2-S, ZH1, ZH6, ZH9, XH7, YX2, WS19, ST6, GL16, NN2, NN1, LZ13, 318 5#, 318 3#, HK2, L3-1, L3-3, M1-3, M2-2, M3-3, M3-5, S1-4, S2-2, S2-4, G2-15, G3-5, G3-7 |
| *Macrotrachela inermis* Donner, 1965 | 19-CD23, 19-CD38, L2-2, L2-5 |
| *Macrotrachela insolita* De Koning, 1947 | 18-C3, ZH1, ZH7, WS19, LZ13 |
| *Macrotrachela induta* Donner, 1951 | CD8 CD7 CD19 CD34, YX1, MW3, LS5, LS4, L1-2, L2-3, L2-5 |
| *Macrotrachela kallosoma* (Schulte, 1954) | CD20 |
| *Macrotrachela libera* Donner, 1949 | 19-CD23 |
| *Macrotrachela multispinosa* Thompson, 1892 | 18-C3, ZH1, ZH2, ZH4, ZH5, ZH8, ZH9, WLD1, QA3, GY1, BM5, L1-1, L1-2, L1-3, L1-4, L1-5, L2-3, L2-5, L3-2, L3-5, M1-1, M1-3, M1-4, M1-5, M2-1, M2-2, M2-3, M2-4, M3-1, M3-2, M3-3, M3-4, M3-5, S2-1, S2-2, S2-5, S3-3, S3-4 |
| *Macrotrachela multispinosa multispinosa* Thompson, 1892 | CD8 CD7 CD21 CD4 CD36 CD33 CD16 CD37 CD3, CS1, CS2, CS3, ST3, ST4, GL2, GL3, GL4, 318 4# |
| *Macrotrachela multispinosa brevispinosa* (Murray, 1908) | CD21 CD33 CD29 CD37 CD3, CS1, XH16, XH27, XH1, YX2, WS7, GL14, GL1, GL6, NN1, GL9, WZ1, KD6, LZ17, KD2, HK3 |
| *Macrotrachela multispinosa flagellata* Bartoš, 1951 | CD29, XH16, XH27 |
| *Macrotrachela nixa* Donner, 1962* | GL12 |
| *Macrotrachela nana* (Bryce, 1912) | CD20, L3-3 |
| *Macrotrachela pacifica* (Murray, 1911)* | S1-1 |
| *Macrotrachela pinnigera* (Murray, 1908)* | GL10, L1-3 |
| *Macrotrachela papillosa* Thompson, 1892 | CD3, FS1, FS2 |
| *Macrotrachela plicata plicata* (Bryce, 1892) | CD4 |
| *Macrotrachela quadricornifera* Milne, 1886 | 19-CD27, 18-C3, CD7 CD36 CD34, YX2, MW6, WS6, WLD6, WLD7, QA3, ST4, GL1, GL3, GL4, GL6, GL7, NN1, WZ1, WZ2, WZ3, KD1, 318 4#, BM10, HK3, HK2, L1-1, L1-2, L1-4, L2-1, L2-2, L2-4, L3-2, L3-3, L3-5, M1-5, M2-1, M3-3, S2-4, S3-4, S3-5, G2-5 |
| *Macrotrachela quadricornifera rigida* Milne, 1916 | CD24, MW6, MW10 |
| *Macrotrachela quadricornifera loricata* Donner, 1965 | CD4, YX2 |
| *Macrotrachela quadricornifera vanoyei* Schepens,1954 | CD34 |
| *Macrotrachela quadricornifera ligulata* Bērziņš, 1950* | GY1 |
| *Macrotrachela timida* Milne, 1916 | CD22 CD33 CD16, XH16, XH19, XH7, XH1, XH19, YX2, GL5, GL8, BM8, KD1, HK2, L1-3, L1-4, L1-5, L2-3, L2-4, L3-3, L3-5, M1-4, M3-2, M3-4, M3-5 |
| *Macrotrachela timida inquies* Milne, 1916 | CD7 |
| *Macrotrachela timida timida* Milne, 1916 | CD33 CD16 CD17, 10-BY2-B |
| *Philodina acuticornis* Murray, 1902 | WS19, WLD1, GL14, L2-3, L2-4, L3-4, L3-5, M1-2, M2-1, M2-3 |
| *Philodina citrina* Ehrenberg, 1830 | 19-CD9, 18-C2, WZ2, M1-1, M1-5, W1-3, W1-4, W2-4, W2-5 |
| *Philodina clypeata* Song & Lee, 2020 | CD22 CD36 CD2, 8-BY1-S, L1-2, L3-1, L3-2, S1-4 |
| *Philodina childi* Milne, 1916 | CD24 |
| *Philodina chinensis* sp. nov. | ST4, LS7, BM8 |
| *Philodina grandis* Milne, 1916 | CD29, 10-BY2-S, L1-4 |
| *Philodina indica* Murray, 1906 | XH16, GL11 |
| *Philodina megalotrocha* Ehrenberg, 1832 | 19-CD33, 18-C1, 10-BY1-B, W1-1, W1-2, W1-3, W1-4, W2-1, W2-2, W2-3, W2-4, W2-5, W3-1, W3-2, W3-3, W3-4, W3-5 |
| *Philodina nemoralis* Bryce, 1903 | CD8, LZ10 |
| *Philodina nitida* Milne, 1916 | GL5 |
| *Philodina plena* (Bryce, 1894) | 19-CD38, 19-CD39, CD8 CD7 CD26 CD36 CD15 CD16 CD24 CD29, FS1, GS3, ST3, GL13, GL14, GL7, NN2, WZ1, LZ13, LZ14, LS5, BM5, BM6, BM8, 318 5#, L1-2, L2-4, L3-1, L3-2, L3-4, L3-5 |
| *Philodina proterva* Milne, 1916 | CD8, FS1, GL10, GL2, LS9, L2-5, M3-1 |
| *Philodina parvicalcar* De Koning, 1947 | BM6, LZ10 |
| *Philodina rapida* Milne, 1916 | CD8 CD15, 9-BY3-S, 10-BY3-S, ZH1, ST3, ST6, L1-3, L1-5 |
| *Philodina rugosa* Bryce, 1903 | CD20 CD10, CS1, XH27, WS3, WS7, QA8, GL15, GL5, GL6, GL17, HK4 |
| *Philodina roseola* Ehrenberg, 1832 | LZ5, L2-4 |
| *Philodina scabra* Milne, 1916 | GL3 |
| *Philodina tranquilla* Wulfert, 1942 | CD33 CD15, SZ2 |
| *Philodina vorax* (Janson, 1893) | CD19, CS1, XH1, XH7, GS3, WS7, ST3, ST4, NN2, 318 5#, BM6, LS3, BDL2, L3-2, L3-5, M1-1, M1-2, M1-4, M1-5, M2-3, M2-5, M3-1, M3-3, M3-4, M3-5, S1-1, S1-2 |
| *Mniobia magna* (Plate, 1889) | CD4, WLD6, ST6, LS7 |
| *Ceratotrocha cornigera* (Bryce, 1893)* | DX4, 318 5# |
| *Philodinavus paradoxus* (Murray, 1905)* | JNU1, NA5 |

*: New records of bdelloid rotifers from China.
